# EEPD1 promotes repair of oxidatively-stressed replication forks

**DOI:** 10.1093/narcan/zcac044

**Published:** 2023-01-18

**Authors:** Aruna S Jaiswal, Hyun-Suk Kim, Orlando D Schärer, Neelam Sharma, Elizabeth A Williamson, Gayathri Srinivasan, Linda Phillips, Kimi Kong, Shailee Arya, Anurag Misra, Arijit Dutta, Yogesh Gupta, Christi A Walter, Sandeep Burma, Satya Narayan, Patrick Sung, Jac A Nickoloff, Robert Hromas

**Affiliations:** Division of Hematology and Medical Oncology, Department of Medicine and the Mays Cancer Center, University of Texas Health Science Center, San Antonio, TX 78229, USA; Center for Genomic Integrity, Institute for Basic Science, Ulsan, Republic of Korea; Center for Genomic Integrity, Institute for Basic Science, Ulsan, Republic of Korea; Department of Biological Sciences, School of Life Sciences, Ulsan National Institute of Science and Technology, Ulsan, Republic of Korea; Department of Environmental and Radiological Health Sciences, Colorado State University, Fort Collins, CO 80523, USA; Division of Hematology and Medical Oncology, Department of Medicine and the Mays Cancer Center, University of Texas Health Science Center, San Antonio, TX 78229, USA; Division of Hematology and Medical Oncology, Department of Medicine and the Mays Cancer Center, University of Texas Health Science Center, San Antonio, TX 78229, USA; Division of Hematology and Medical Oncology, Department of Medicine and the Mays Cancer Center, University of Texas Health Science Center, San Antonio, TX 78229, USA; Division of Hematology and Medical Oncology, Department of Medicine and the Mays Cancer Center, University of Texas Health Science Center, San Antonio, TX 78229, USA; Department of Biochemistry and Structural Biology, University of Texas Health Science Center, San Antonio, TX 78229, USA; Department of Biochemistry and Structural Biology, University of Texas Health Science Center, San Antonio, TX 78229, USA; Department of Biochemistry and Structural Biology, University of Texas Health Science Center, San Antonio, TX 78229, USA; Department of Biochemistry and Structural Biology, University of Texas Health Science Center, San Antonio, TX 78229, USA; Department of Cell Systems and Anatomy, University of Texas Health Science Center, San Antonio, TX 78229, USA; Department of Biochemistry and Structural Biology, University of Texas Health Science Center, San Antonio, TX 78229, USA; Department of Neurosurgery, University of Texas Health Science Center, San Antonio, TX 78229, USA; Department of Anatomy and Cell Biology, University of Florida, Gainesville, FL 32610, USA; Department of Biochemistry and Structural Biology, University of Texas Health Science Center, San Antonio, TX 78229, USA; Department of Environmental and Radiological Health Sciences, Colorado State University, Fort Collins, CO 80523, USA; Division of Hematology and Medical Oncology, Department of Medicine and the Mays Cancer Center, University of Texas Health Science Center, San Antonio, TX 78229, USA

## Abstract

Unrepaired oxidatively-stressed replication forks can lead to chromosomal instability and neoplastic transformation or cell death. To meet these challenges cells have evolved a robust mechanism to repair oxidative genomic DNA damage through the base excision repair (BER) pathway, but less is known about repair of oxidative damage at replication forks. We found that depletion or genetic deletion of EEPD1 decreases clonogenic cell survival after oxidative DNA damage. We demonstrate that EEPD1 is recruited to replication forks stressed by oxidative damage induced by H_2_O_2_ and that EEPD1 promotes replication fork repair and restart and decreases chromosomal abnormalities after such damage. EEPD1 binds to abasic DNA structures and promotes resolution of genomic abasic sites after oxidative stress. We further observed that restoration of expression of EEPD1 via expression vector transfection restores cell survival and suppresses chromosomal abnormalities induced by oxidative stress in EEPD1-depleted cells. Consistent with this, we found that EEPD1 preserves replication fork integrity by preventing oxidatively-stressed unrepaired fork fusion, thereby decreasing chromosome instability and mitotic abnormalities. Our results indicate a novel role for EEPD1 in replication fork preservation and maintenance of chromosomal stability during oxidative stress.

## INTRODUCTION

One of the greatest threats a cell faces is the continuous damage of its DNA by reactive oxygen species (1–3). Cells have evolved a robust repair pathway to repair oxidative DNA damage, the base excision repair (BER) pathway (1,2,4). Oxidative DNA damage is usually repaired without genetic consequence, but that may not be true during DNA replication, where oxidative damage can stall replication forks and ultimately lead to their collapse or fusion (5–7). The obligate single-strand (SS) nicked BER intermediate can convert into a double-strand break (DSB) when encountered by a replication fork, causing fork collapse (8–13). Thus, collapsed forks represent a far greater danger to the cell than oxidatively damaged nucleotides elsewhere in the genome. Collapsed replication forks can degrade, leading to cell death or they may fuse, leading to aneuploidy and neoplastic transformation, or cell senescence and aging (8–13).

BER is a coordinated process that repairs DNA damage from oxidation, alkylation, and deamination. BER has four core steps, (i) base damage recognition and removal, (ii) DNA strand cleavage at the ensuing abasic site (also commonly termed apurinic/apyrimidinic or AP sites), (iii) DNA synthesis to replace the damaged nucleotide and removal of displaced parent DNA flap intermediate and (iv) DNA ligation. BER has two sub-pathways, short-patch (SP) and long-patch (LP) BER. Each sub-pathway can be initiated by at least 11 glycosylases that catalyze the excision of damaged bases to generate AP sites (1–4). After the damaged base is removed, the next step is endonucleolytic strand cleavage 5′ of the abasic site, most commonly by APE1 (1–4,14,15). There are two main classes of AP endonucleases, class I cleaves 3′ to the abasic residue and class II cleaves 5′ to the abasic site, the canonical example of which is APE1. SP-BER employs a DNA deoxyribophosphodiesterase to excise dRP residues, and in LP-BER 5′ flap DNA structures are cleaved by the structure-specific endonuclease, flap endonuclease 1 (FEN1), then DNA polymerase β (POL β) fills the single-strand gap (1–4). In SP-BER, the XRCC1/DNA ligase 3 (LIG3) complex catalyzes the DNA ligation step, while LP-BER uses DNA ligase 1 (LIG1) (2,16). However, if the AP-site is modified and is resistant to the dRP-lyase activity of Pol β, an alternative polymerase may displace a segment of single-stranded DNA containing the lesion. The displaced strand is then cleaved by the structure-specific enzyme flap endonuclease 1 (FEN1) and the resulting nick is ligated, most likely by DNA ligase I (1–4). Many of the components of BER also participate in DNA replication, indicating mechanistic links between BER and replication (1–7).

Repair of oxidative damage is best characterized in non-replicating DNA (1–6), but there is evidence that APE1 and NEIL1/3 are involved in the repair of oxidatively stressed replication forks (17–19). NEIL1,2,3 are DNA glycosylases that have a broad range of substrate specificity, and can excise either damaged or ring-opened oxidized purines and pyrimidines. APE1 is a multifunctional protein involved in the protection of cells from oxidative stress via its DNA repair, redox and transcriptional regulatory activity (14,15). However, there is evidence of an alternative, APE1-independent BER pathway. For example, there are cancers that do not express APE1 or express APE1 at low levels, yet can still replicate DNA and survive without difficulty (20–23). APE1 is poorly expressed in aging organs such as the liver and central nervous system yet the organism still survives (24–27). Some cancer cell lines can maintain their proliferative potential despite APE1 deletion (25,26). These data suggest the existence of APE1 backup pathways.

DNA single-strand breaks (SSBs) are among the most common DNA lesions in cells. In neuronal cells, oxidative stress is a major source of SSBs. SSBs accumulate at a higher frequency in neuronal cells compared with other postmitotic cells because of the relatively high level of oxidative metabolism in the central nervous system (24). It has also been observed that there are some abasic DNA structures that APE1 cannot cleave (28,29). These structures can stall the replication forks and result in unwanted consequences for actively dividing cells. If oxidatively-stressed replication forks are not repaired and restarted in a timely manner, they may degrade, resulting in apoptosis, or fuse to other DSB ends, resulting in chromosomal translocations which can cause mitotic catastrophe, or if the cell survives, aneuploidy (9–13).

Stressed replication forks require a free DNA end to load exonucleases to initiate 5′ end resection complexes for homologous recombination (HR) repair and restart of the fork (30–33). This DS free end can either be generated from fork reversal to a chicken foot structure, or endonucleolytic cleavage of the fork structure itself (34–36). Fork cleavage can be accomplished by 3′ cleavage by MUS81 or 5′ cleavage by EEPD1 (30,35,36). Fork cleavage is a risk for the cell because it creates a one-ended DSB and if not repaired and restarted in timely manner, forks can degrade or fuse to other DSB ends leading to chromosomal rearrangements or missegregation, aneuploidy, neoplastic transformation, cell senescence, or cell death (8–13) Thus improper processing of oxidatively stressed replication forks represent a far greater danger to the cell than oxidative damage elsewhere in the genome.

We previously reported that EEPD1 initiates BRCA1-independent HR repair of stressed replication forks in cells treated with hydroxyurea (30,35,37–39). EEPD1 cleaves the lagging parental strand of the stalled replication fork structure at the junction of the single-strand and double-strand DNA and loads EXO1 onto that site (32,35). In the present study, we discovered that EEPD1 has 5′ abasic endonuclease activity, similar to APE1 (14,15). We therefore hypothesized that EEPD1 contributes to the resolution of oxidative lesions similar to APE1, serving as an alternative to APE1 in BER, especially at replication forks. Such an alternative BER pathway at replication forks may be critical to maintain genome stability when APE1 is not expressed or its function is saturated due to excessive oxidative damage. We demonstrate here that EEPD1 can resolve genomic oxidative damage and promote repair and restart of oxidatively-stressed replication forks. We found that EEPD1-mediated repair of these lesions at the replication fork is critical to prevent chromosomal instability. Cells with EEPD1 defects develop chromosomal fusions at oxidatively-damaged replication forks that led to retained chromosomes after mitosis, revealed as gross nuclear abnormalities such as nuclear bridges and micronuclei (12,30,40). Together the results indicate that EEPD1 functions in an alternative BER-like pathway to repair oxidative DNA damage at replication forks, which is important for genomic stability.

## MATERIALS AND METHODS

### Cell lines and culture

Human cell lines HCT116 (colon cancer), 22Rv1 (prostate cancer), HEK293 (embryonic kidney), HeLa (cervical cancer), U-87 (glioma) and A549 (lung cancer) were obtained from the American Type Culture Collection (ATCC, Manassas, VA, USA). HeLa control, HeLa EEPD1 CRISPR knockout (KO), HEK293, U-87 and A549 cells were grown in DMEM medium supplemented with 10% fetal bovine serum albumin (FBS; Atlanta Biologicals, Atlanta, GA, USA), 100 U/ml of penicillin and 100 μg/ml of streptomycin. HCT116 cells were grown in McCoys5a medium and 22Rv1 cells were grown in RPMI medium supplemented with 10% FBS, 100 U/ml of penicillin, and 100 μg/ml of streptomycin.

### SiRNA depletion of EEPD1, APE1, and metnase

EEPD1 was transiently depleted in HCT116 and other cells using small interfering RNA (siRNA) and Lipofectamine RNAiMAX transfection reagent (Life Technologies, USA). We used SMARTpool ON-TARGETplus and a non-target pool as a Scr-siRNA control. SMARTpool and On-TARGETplus siRNA for EEPD1 and Scr-siRNA control were purchased from Dharmacon (Lafayette, CO, USA). The siRNA sequences for EEPD1 were: GGACUGACCUUCACCGCCA, CUGAGAAGCCCUCGAGUCA, GGAAGUUGACCUCGGGGUA, and UGCGAGAGGUGGUGUGCAU; for APE1: CAAAGUUUCUUACGGCAUA, GAGACCAAAUGUUCAGAGA, CUUCGAGCCUGGAUUAAGA and UAACAGCAUAUGUACCUAA; and for Metnase: CAAGUGUUCAAGACGCAUA, CCGUAGAAAAGUCGAACAU, CUUGGAAUUUAUACCGAAA, and UGACAACCGGCGACGAUCA. Briefly, a day before transfection, HCT116 or HEK293 cells were seeded at a density of 1.5×10^5^ cells per well in 6-well plates. After 24 h the DNA:lipid complex was prepared by gently mixing Lipofectamine RNAi-Max and siRNA (30 nM) (30,41) and incubated for 30 min at room temperature. Cells were washed once with Opti-MEM medium and fresh Opti-MEM medium was added to completely remove serum and antibiotics from cultured cells. The DNA:lipid complex was slowly added to the cells, and cells were incubated for 6–8 h at 37°C in 5% CO_2_. Eight hours after transfection, 0.5 ml of Opti-MEM medium containing 30% serum was added to each well, and cells were incubated overnight. The DNA:lipid complex was removed 24 h post-transfection and replaced with a complete growth medium. Cells were harvested 48 h post-transfection. Cell lysates were prepared, and protein levels were checked for the repression of target proteins by western blotting as described below. Genetic deletion of EEPD1 in HeLa cells was described previously (39).

### Clonogenic cell survival

Clonal cell survival of HCT116, HEK293, 22-Rv1, U-87, A549 and HeLa cells deleted of EEPD1 was determined by plating control or test cells at a density of 5×10^2^ cells per well in 6-well dishes in triplicate (30). After attachment, cells were treated with different concentrations of H_2_O_2_ or methyl methanesulfonate (MMS) for 24 h. Medium containing H_2_O_2_ or MMS was removed, cells were washed with a fresh complete medium and supplemented with fresh medium. Cells were allowed to grow for 7–10 days to form visible colonies (>50 cells). Colonies were stained with 1% crystal violet in methanol, dried, and counted using ImageJ software (US National Institute of Health, Bethesda, MD, USA) (42). The surviving fraction was calculated by normalizing against the colony numbers achieved for untreated wells. All experiments were repeated at least three times and the data shown are the mean ± SE. Representative images of clonogenic survival assays are shown in Supplementary Figure S1.

### Oligonucleotides and antibodies

All oligonucleotides with and without an abasic site mimic (3-hydroxy-2-hydroxymethyltetrahydrofuran, termed THF) for BER, EMSA and biochemical fluorescence polarization were custom synthesized from Sigma-Genosys (Woodlands, TX, USA). All oligomers were purified by polyacrylamide gel electrophoresis, reconstituted in TE buffer pH 8.0 (10 mM Tris, pH 8.0 and 1 mM EDTA, pH 8.0), and quantitated spectrophotometrically. Sequences of oligonucleotides used in this study are listed in Supplementary Table S1. Dilutions and sources of all antibodies used in this study are listed in Supplementary Table S2. Antibody for EEPD1 was mouse monoclonal custom-produced at the Interdisciplinary Center for Biotechnology Research Core Facility, University of Florida, Gainesville, FL, USA (30).

### Oligonucleotide annealing and radiolabeling

Equimolar amounts of complementary oligonucleotides were annealed in 0.05 ml containing 10 mM Tris, pH 7.5, 1 mM EDTA, 100 mM KCl. Solutions were heated to 85°C for 10 min and allowed gradually to cool to room temperature and later used for labeling. Annealed oligos were stored at -20°C. Annealed double-strand oligos were labeled with [γ-^32^P]ATP at the 5′-end using T4 polynucleotide kinase using a standard protocol (41). The labeled oligomers were purified using Nick columns as described by the manufacturer (GE Healthcare Biosciences, Pittsburgh, PA), quantitated, and stored at −20°C until use. Oligos for BER assays were labeled on sense-strands and then annealed to their corresponding complementary oligos (Oligo A and C for nuclease activity; Oligo B and C for SP-BER activity, Supplementary Table S1). Nucleotide sequences for SP-BER substrates contained one uracil residue at nt 24 (Oligo B, Supplementary Table S1) as described (41). T4-polynucleotide kinase and uracil DNA glycosylase were purchased from New England Biolabs. Radionuclide [γ-^32^P]ATP was purchased from Perkin Elmer, Inc. (Boston, MA, USA).

### Purification of APE1, EEPD1, POL β and XRCC1/LIG3

Human recombinant hexahistidine-tagged APE1 was overexpressed in BL21 (DE3) cells and purified to homogeneity according to our published protocols (41,43), and recombinant FLAG-tagged EEPD1 was purified to homogeneity from HEK293 cells using affinity chromatography (Supplementary Figure S2A–D) as we described earlier (35). We acquired purified human POL β from the late Samuel Wilson (NIEHS, Research Triangle Park, NC, USA) and purified XRCC1/LIG3 complex from Alan Tomkinson (University of New Mexico, Albuquerque, NM, USA).

### Electrophoretic mobility shift assay

Equimolar quantities of oligonucleotides were annealed for binding experiments and labeled with T4 polynucleotide kinase using standard protocols as we described (35,41). DNA substrates with their complimentary oligos are listed in Supplementary Table S1. For the abasic site oligomer (Oligo I and E; Oligo D and E for abasic DNA structure and linear non-abasic structure; Supplementary Table S1), fork substrate oligomer (Oligos I, F and G, H; Oligos D, F and G, H; Supplementary Table S1) were mixed in equimolar concentration, annealed and labeled at 5′-ends using T4 polynucleotide kinase. DNA-protein binding reactions were assembled in a final volume of 20 μl, containing 20 mM HEPES, pH 7.9, 1 mM dithiothreitol, 30 mM NaCl, 0.03% (v/v) Nonidet *P*-40, 10% (v/v) glycerol, 1 μg of poly (dI-dC) and varying amounts of recombinant EEPD1 protein (100–300 ng 2.5–7.5 pmol). Reactions were initiated by the addition of ^32^P-labeled control oligomer, ^32^P-30-mer F-DNA oligonucleotides, ^32^P-30-mer fork-DNA oligonucleotides, and ^32^P-30-mer Fork with abasic lesion DNA oligonucleotides (44–46). Reactions were allowed to proceed for 20 min at room temperature, and then the entire reaction mixture was loaded directly onto a 5.5% polyacrylamide gel. DNA-bound protein was resolved by non-denaturing gel electrophoresis at 4°C in 1× TBE buffer. For competition experiments, a molar excess (10×, 25× and 50×) of an unlabeled identical competitor oligo was added to the reaction mixture 10 min prior to the addition of the ^32^P-labeled oligo probe. After electrophoresis, the gel was transferred to a blotting sheet (3MM chromatographic paper, Whatman) and dried. DNA-protein complexes were visualized by autoradiography. Experiments were repeated at least three times.

### Equilibrium binding of EEPD1 and APE1 to DNA

Fluorescence polarization assays were used to measure equilibrium dissociation constants (*K*_D_). We annealed equimolar quantities of four DNA oligos (Oligo I, F, G, H; Supplementary Table S1) to generate a fork structure with a fluorescein moiety covalently attached to the 5′-end of sense oligo. A synthetic THF abasic site analog was inserted on the sense strand and annealed with an equimolar quantity of anti-sense strand to generate a double-strand fork structure with an abasic site. Reactions were assembled, and equilibrium binding affinities were measured using a fluorescence polarization-based assay (47). A constant concentration of fluorescein-labeled fork DNA (5 nM) and increasing concentrations of EEPD1 or APE1 protein (0–1000 nM) were mixed with 10 mM HEPES pH 7.5, 50 mM KCl in a 384-well plate. The fluorescence polarization value for each dilution was measured using PHERAstar FS (BMG Labtech, WI, USA) at an emission of 530 nm and excitation at 485 nm. The buffer-corrected values of triplicate runs were used to calculate the *K*_D_ for protein-DNA binding using a simple 1:1 specific binding model. Experiments were repeated at least three times and *K*_D_ values were calculated as the mean of all experiments.

### Isolation of proteins on nascent DNA (iPOND)

IPOND was performed as described with some modifications (30,48,49). At least 40×10^6^ HCT116 cells per sample were labeled with 10 μM EdU for 10 min followed by treatment with 1 mM H_2_O_2_ for 1 h and then released for indicated times. At each time point, cells were fixed with 1% formaldehyde for 15 min at room temperature and then quenched with 1/10 volume of 1.25 M glycine. Formaldehyde fixed and neutralized cells were harvested by scraping and collected by centrifugation at 4°C for 8 min followed by three washes with cold PBS. Cells with EdU-labeled nascent DNA were permeabilized with 0.25% Triton X-100 for 30 min at room temperature in the dark followed by conjugation with biotin-azide by click chemistry for 2–3 h at room temperature in the dark. Briefly, nuclei from cells (40×10^6^) were suspended in PBS buffer, followed by the addition of 0.04 ml biotin azide (10 μM), 0.4 ml sodium ascorbate (10 mM), and 0.08 ml copper sulfate (2 mM), and final volume was adjusted to 4 ml. Contents were quickly mixed and rotated for 2–3 h in the dark. The no-click, negative control was processed by substituting DMSO for biotin-azide. After click chemistry, samples were washed twice with cold PBS. Isolated nuclei were lysed using 0.25% SDS lysis buffer followed by sonication as we described earlier (30) and insoluble debris was removed by centrifugation. Supernatants were collected and proteins bound to EdU-labeled DNA were pulled down with streptavidin-agarose beads (50 μl) and incubated for 16–20 h at 4ºC. Streptavidin-agarose beads were washed once with lysis buffer, once with 1M NaCl, and twice with lysis buffer, and finally suspended in 2× sample buffer. Proteins were eluted in 2× SDS sample buffer at 95ºC for 25 min. Input and IPOND captures were used to detect H3, PCNA, APE1, POL β, FEN1, PARP1, LIG1 and EEPD1 by western blot (Supplementary Table S2). Both si-Scr control and si-EEPD1 depleted samples after treatment with H_2_O_2_ were processed for DNA elution for 8-oxo-dG quantitation. Streptavidin-agarose beads were washed as described above followed by a quick wash with water. Beads were suspended in water or TE buffer and incubated at 65°C for 5 min followed by centrifugation at 1800×g for 3 min. Eluted DNA was collected from the IPOND-captured samples in a separate tube. Eluted DNA was quantitated spectrophotometrically. Normalized DNA was used to estimate 8-oxo-dG levels in both control and EEPD1-depleted samples treated with H_2_O_2_. All iPOND experiments were repeated at least three times. Thymidine chase was performed to verify EEPD1 recruitment to replication forks, similar to the iPOND procedure. Cells were labeled with 10 μM EdU for 10 min and EdU was quickly removed by washing with a prewarmed culture medium. After washing, cells were incubated with fresh medium equilibrated with 10 μM thymidine at 37°C for 0–60 min. Cells were harvested after fixation with 1% formaldehyde and neutralization with 1.25 M glycine for 10 min. Cells were processed similar to iPOND as described above.

### EEPD1 nuclease activity assay

Nuclease activity assays were carried out as described earlier (35,41,50). Standard reactions (25 μl) contained 30 mM Tris, pH 7.5, 30 mM KCl, 5 mM MgCl_2_, 2 mM DTT, 0.01% Nonidet *P*-40, 0.1 mg/ml bovine serum albumin, and APE1 (5 fmol) or varying concentrations of purified recombinant EEPD1 protein (0.4–3 pmol) were added to the tubes on ice. The reaction mixtures were assembled on ice and reactions were initiated by the addition of labeled DNA substrate (Oligomers A and C, Supplementary Table S1). The reaction was incubated at 37°C for 1 h and terminated by the addition of stop buffer (0.4% SDS (w/v), 5 mm EDTA, 1 μg of proteinase K). The DNA was recovered by phenol/chloroform extraction and ethanol precipitation followed by centrifugation. The recovered DNA was washed with cold 70% ethanol, and DNA was dried, suspended in 10 μl of sample loading dye (95% formamide, 1 mM EDTA, 0.1% xylene cyanol and 0.1% bromophenol blue). DNA samples were denatured by heating at 85°C for 5 min and were immediately cooled on ice. Samples (4 μl) were loaded onto a premade and prerun 15% denaturing polyacrylamide with 7 M urea gel for electrophoresis. The gel was dried under a vacuum and autoradiography was used to detect radioactive signals. For non-radioactive oligo detection, DNA on gels was visualized by scanning with a Typhoon Trio Biomolecular Imager (GE Healthcare Bio-Science Corporation, Piscataway, NJ, USA), at excitation of 526 nm and emission at 488 nm. Digital images were acquired and quantified using ImageQuant TL (GE Healthcare Bio-Science Corporation) software for densitometric analysis of the efficiency of cleavage. Graphical data are the mean ± SE of >3 experiments each from distinct protein preparations.

### *In vitro* reconstitution of SP-BER

*In vitro* SP-BER assays were assembled on ice as we described previously (41,50). The SP-BER substrate DNA was U DNA (Oligo B and C, Supplementary Table S1) and was treated with uracil DNA glycosylase to create an abasic site. U DNA substrate was precleaved by the nuclease activity of EEPD1 for 20 min. This precleaved substrate was used in reconstituted SP-BER assays. The BER reaction mixture was assembled on ice by the addition of 30 mM Tris, pH 7.5, 30 mm KCl, 8 mm MgCl_2_, 1 mm dithiothreitol, 100 μg/ml bovine serum albumin, 0.01% Nonidet *P*-40, 0.5 mm ATP, and 10 μm each of dATP, dCTP, dGTP, dTTP in a final reaction volume of 25 μl. SP-BER reaction was initiated by the addition of 100 fmol of labeled 43-mer U-DNA substrate, 2.5 fmol of APE1 or 3 pmol of purified recombinant EEPD1 protein, 25 fmol of Pol β, and 25 pmol each of XRRC1/LIG3. The reaction mixture was incubated at 37°C for 60 min. Reactions were terminated with stop buffer (0.4% (w/v) SDS, 5 mm EDTA, 1 μg of proteinase K) and incubated at 37°C for an additional 20 min. The DNA was recovered by phenol/chloroform extraction and ethanol precipitation. The recovered DNA was washed with cold ethanol (70%, *v/v*), dried, and suspended in 10 μl of sample loading dye. The reaction products were resolved on a 15% acrylamide and 7 M urea gel. DNA on the gel was visualized by scanning on Typhoon Trio Biomolecular Imager (GE Healthcare Bio-Science Corporation, Piscataway, NJ, USA), at excitation of 526 nm and emission at 488 nm. Digital images were acquired and quantified using ImageQuant TL (GE Healthcare Bio-Science Corporation, Piscataway, NJ, USA) software. Each BER assay was performed 3–5 times using distinct protein preparations of all components. The repair efficiency was quantified by densitometric analysis of the ligated full-length product compared to the cleaved products. Graphical data are the mean ± SE of all experiments.

### Single cell DNA electrophoresis (alkaline comet) assay

Alkaline single-cell electrophoresis assays for DNA damage and repair were performed using the Comet Assay kit (Trevigen, Gaithersburg, MD, USA) as we described (30). Briefly, HCT116 cells were transfected with siRNA-Scr control or siRNA-EEPD1 and treated with 250 or 500 μM H_2_O_2_ for 1 h. Cells were protected from light, trypsinized, counted, and adjusted to 1×10^5^ cells per ml in PBS, and 2000 cells were seeded into each well on a Comet slide. After electrophoresis, DNA from cells was visualized using SYBR green dye. Images were captured using a fluorescence microscope (Zeiss Axiovert 200M, Thornwood, NY) at 10X magnification. Tail length was calculated using the OpenComet plug-in on ImageJ software. All experiments were repeated at least three times and the data shown are the mean ± SE.

### AP site quantitation

AP sites in genomic DNA were quantified as we described with some modifications (41,50,51). Briefly, genomic DNA from HCT116 cells was isolated using a Mammalian Genomic DNA Isolation Kit (Qiagen, USA). Two to five microgram of genomic DNA was incubated with 1 mM aldehyde reactive probe (ARP) (Dojindo Molecular Technology, MD, USA) in 100 μl of PBS at 37ºC for 10 min to label AP sites with aldehyde reactive probe in genomic DNA. After ARP labeling, DNA was ethanol precipitated and recovered by microcentrifugation at room temperature. ARP-conjugated DNA was dissolved in TE buffer (10 mM Tris–HCl, 1 mM EDTA, pH 7.5) and quantified. One microgram of 95°C heat-denatured ARP-conjugated DNA was slot-blotted onto a positively charged nylon membrane (Amersham Corp., Piscataway, NJ, USA) and dried. The membrane was soaked in 5× SSC (0.75 M NaC1, 0.075 M trisodium citrate) at 37°C for 15 min, and DNA was cross-linked to the membrane by incubating in an 80°C vacuum oven for 2 h. The dried membrane was preincubated with 10 ml of Tris-NaCl buffer containing BSA (20 mM Tris–HCI (pH 7.5), 0.1 M NaCl, 1 mM EDTA, 0.5% casein, 0.25% BSA, and 0.1% Tween 20) at room temperature for 1 h, then incubated with streptavidin-conjugated horseradish peroxidase (Invitrogen, Carlsbad, CA, USA) for 30–45 min at room temperature. After rinsing the membrane twice with washing buffer (0.26 M NaCl, 1 mM EDTA, 20 mM Tris–HCl, and 0.1% Tween 20, pH 7.5) for 5 min, the AP-sites conjugated to ARP were visualized on the membrane with enhanced chemiluminescence (ECL) reagents. Signals for abasic sites were captured on X-ray film by enhanced chemiluminescence. AP sites were quantified based on densitometric comparison to the standard curve of known amounts of AP sites in DNA. All experiments were repeated at least three times and data are presented as mean ± SE.

### DNA fiber assays

DNA fiber analysis was carried out as we described (30,35,52). Briefly, cells were grown in 6-well plates (2×10^5^ cells/well). EEPD1 or APE1 were depleted by siRNA as described above, or HeLa cells with wild-type EEPD1 or EEPD1 KO were used. After 48 h post-transfection cells were pulse-labeled with 100 μM IdU and incubated at 37°C for 20 min. After washing with fresh medium, cells were treated with 1 mM H_2_O_2_ for 15 min or mock-treated. Medium was replaced with fresh medium containing 300 μM CldU, and cells were further incubated for indicated times at 37°C. Cells were harvested, washed with PBS, and resuspended in PBS at 2×10^5^ cells per ml. DNA fibers were processed using a FiberComb molecular combing system (GenomicVision, Bagneux, FR). Cells were mixed with low-melting agarose and agarose plugs were made. Agarose plugs were chilled at 4°C for 30 min to solidify the agarose. Each plug was mixed with 200 μl 0.5M EDTA, 25 μl sarkosyl, and 50 μl proteinase K and incubated at 50°C for 18 h. Plugs were washed three times with TE buffer (10 mM Tris pH 8, 1 mM EDTA) at room temperature and placed in a reservoir containing 1 ml of MES buffer (pH 5.5). These reservoirs containing plugs were incubated at 65°C for 30 min to melt the agarose. The melted agarose was digested with 2 μl of agarase (New England Biolabs) at 42°C for 14–18 h. After digestion with agarase, these reservoirs were stored at 4°C for 2–3 days before processing for DNA fibers. DNA fibers were processed on slides (GenomicVision, Bagneux, FR) using the FiberComb molecular combing system. The newly synthesized CldU and IdU tracks were labeled (for 2.5 h in the dark, at RT) with antibodies recognizing CldU and IdU, followed by 1 h incubation with secondary antibodies at RT in the dark. Slides were mounted in PermaFluor aqueous self-sealing mounting medium (Thermo-Fisher Scientific, MA, USA), and images of DNA fibers were captured with a confocal Olympus FV1000D scanning microscope (Olympus America Inc., Center Valley, PA, USA). DNA fiber images were analyzed using ImageJ software. At least 200 tracks were quantified for each experiment. All experiments were repeated at least three times and data were expressed as mean ± SE.

### Replication fork fusion and fork degradation assays

A modified DNA fiber analysis procedure was performed to assess the fate of unrepaired replication forks after oxidative stress (30,35,52–55), including fusion with another unrepaired fork, or degradation. When both IdU and CldU are present at the same time, the amount of incorporation of one versus the other is governed by the frequency of the cognate opposite base for pairing. Thus, the pattern of green:red within the same fiber when both labels are present at the same time is unique for each replication fork. This characteristic can be used to code individual replication forks. In addition, new replication forks moving away from the same origin exhibit approximately the same length of labeled fibers because they have been progressing for the same length of time. Inappropriate fusion of two collapsed replication forks would therefore result in asymmetrically-labeled and asymmetrically-sized forks adjacent to each other on a single DNA fiber. It is recognized that such unrepaired replication fork fusion events will be a subset of such asymmetric fibers, as they can also reflect rescue of a stalled fork by an adjacent fork.

To measure unrepaired fork fusion events, HCT116 cells were grown in 6-well dishes (1.5×10^5^ cells/well) and EEPD1 was depleted by siRNA. Forty-eight hours post-transfection cells were pulse-labeled with 25 μM CldU for 30 min at 37°C followed by the addition of 25 μM IdU without removing CldU. Cells were incubated at 37°C for 30 min, washed with a fresh medium, and incubated with a medium containing 1 mM H_2_O_2_ at 37°C for 1 h. Cells were washed with fresh medium and allowed to grow for 2 h. Cells were trypsinized and collected after centrifugation. Cells were processed for DNA fiber analysis as described above, with the modifications that CIdU was stained red and IdU green, and that fibers were stained with DAPI to verify that adjacent tracks were on the same fiber. Images of the fraction of two adjacent asymmetrically-labeled and asymmetrically-sized forks on the same fiber compared to total forks were captured with a Nikon Eclipse Ti microscope (Nikon, NY, USA) and analyzed with ImageJ software. Representative images from >270 captured images in each condition of four independent experiments are shown. Data are the mean ± SE from four independent experiments.

Fork degradation was measured by the ratio of the newly synthesized ldU fiber length to the previously labeled CIdU fiber length when continuously exposed to H_2_O_2_ (53–56). Degradation proceeds from newly synthesized DNA to older nascent DNA, which results in the shortening of the second label, ldU before the first label, CIdU. The method is similar to the DNA fiber analysis described above, with minor modifications. Here, the CIdU was stained red and IdU green. After 20 min at 37°C, 25 μM CIdU-containing medium was washed off and replaced with a fresh medium containing 125 μM ldU for 20 min at 37°C. The medium was replaced with a medium containing 2.5 mM H_2_O_2_ for 1 h at 37°C. Cells were trypsinized and processed as above for DNA fiber analysis. Slides were mounted in PermaFluor aqueous, self-sealing mounting medium (Thermo-Fisher Scientific), and DNA fibers were visualized by confocal microscopy and analyzed as above. Data were from four independent experiments with >200 tracks scored per condition in each experiment and expressed as mean ± SE.

### Micronuclei quantitation

Control and siRNA EEPD1-depleted HCT116 cells and HeLa control or CRISPR/Cas9 EEPD1-deleted cells (39) were exposed to H_2_O_2_ and nuclei stained with DAPI as described (12,30). Immunofluorescent confocal microscopy was used to count cells with micronuclei compared to cells with normal nuclei. Greater than 300 cells were imaged per experiment, and each experiment was performed three times. The data shown are the mean ± SE.

### Statistical analysis

All experiments were performed at least 3 times and no experimental data was excluded from the statistical analysis. Statistical analysis was performed using a two-way analysis of variance (ANOVA) by using SigmaPlot 13 (Systat Software, San Jose, CA) or two-tailed *t*-tests by using Prism software (GraphPad, San Diego, CA). The criterion for statistical significance was *P* ≤ 0.05. For western blotting data, band intensities were measured using ImageJ (42) and normalized to the loading control (β-actin or eEF2).

## RESULTS

### EEPD1 promotes cell survival after oxidative DNA damage

**Figure 1.**
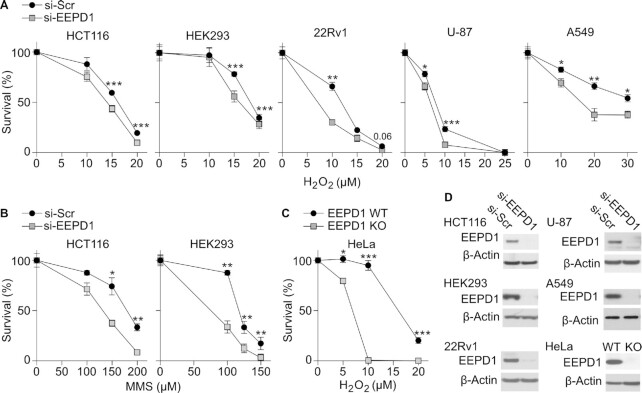
EEPD1 promotes cell survival after oxidative and alkylating DNA damage. (**A–C**) Graphs of clonal cell survival after si-EEPD1 transfection and 24 h exposure to indicated concentrations of H_2_O_2_ or MMS to induce damage repaired by BER.**(**C) Cell survival of HeLa WT or EEPD1 KO. For panels **A-C**, data are means ± SE of three independent experiments. Statistics were calculated with two-tailed t-tests; in this and all subsequent figures, * *P* < 0.05, ** *P* < 0.01, *** *P* < 0.001. (**D**) Western blots of EEPD1 in five cell lines with or without EEPD1 depletion and in HeLa WT and EEPD1 KO cells; β-actin served as a loading control.

To understand the role of EEPD1 in cell survival after exposure to various DNA-damaging agents, we performed colony formation assays using cells with or without EEPD1 depletion. We subjected HCT116, HEK293, 22Rv1, U87 and A549 cells with and without EEPD1 depletion to oxidative stress by treating cells with different concentrations of H_2_O_2_ for 24 h. We found that EEPD1 depletion sensitizes cells to DNA damage repaired by BER in all tested cell lines (Figure 1A–C and Supplementary Figure S1) when normalized to survival of cells with only EEPD1 depletion. While all cells had a decrease in survival to oxidative/alkylative damage after EEPD1-depletion, U-87 glioma cells, which arose in a hypoxic environment and are particularly sensitive to H_2_O_2_ when EEPD1 is depleted (Figure 1A–C and Supplementary Figure S1). Normalized to colony formation with EEPD1 depletion alone, there was up to a 2.7-fold decrease (U-87) in clonal survival at the highest H_2_O_2_ concentration (Figure 1A, B) and up to a 3.2-fold decrease in survival at the highest concentration of MMS (Figure 1B). We also monitored the growth of both HCT116 control and HCT116 after EEPD1 depletion, APE1 depletion and double APE1/EEPD1 depletion by MTT s and found that there was no significant difference in the growth rates of these cells. We further validated our results by using HeLa EEPD1-deleted cells, which were almost completely killed by 24 h treatment with 10 μM H_2_O_2_ (∼1% survival) while 94% of HeLa control cells survived this treatment and formed colonies (Figure 1C, D). EEPD1 protein levels were confirmed in each cell line used in clonogenic survival assays (Figure 1D). We also assessed EEPD1 expression in a panel of fifteen untreated cell lines by western blot analysis and found that EEPD1 is expressed in all cancer and non-cancer cell lines, and thus could have a role in DNA repair that crosses tissue specificities (Supplementary Figure S2E). We also observed that oxidative insult with H_2_O_2_ increased EEPD1 expression in HCT116 cells (Supplementary Figure S2F). We further tested whether restoration of EEPD1 expression can restore colony survival in EEPD1-depleted cells after oxidative stress. Indeed, transduced expression of wild-type EEPD1 in si-EEPD1 depleted cells restored colony survival to levels seen with control si-Scr HEK293 cells (Supplementary Figure S3).

### EEPD1 promotes repair and restart of oxidatively-stressed replication forks

**Figure 2.**
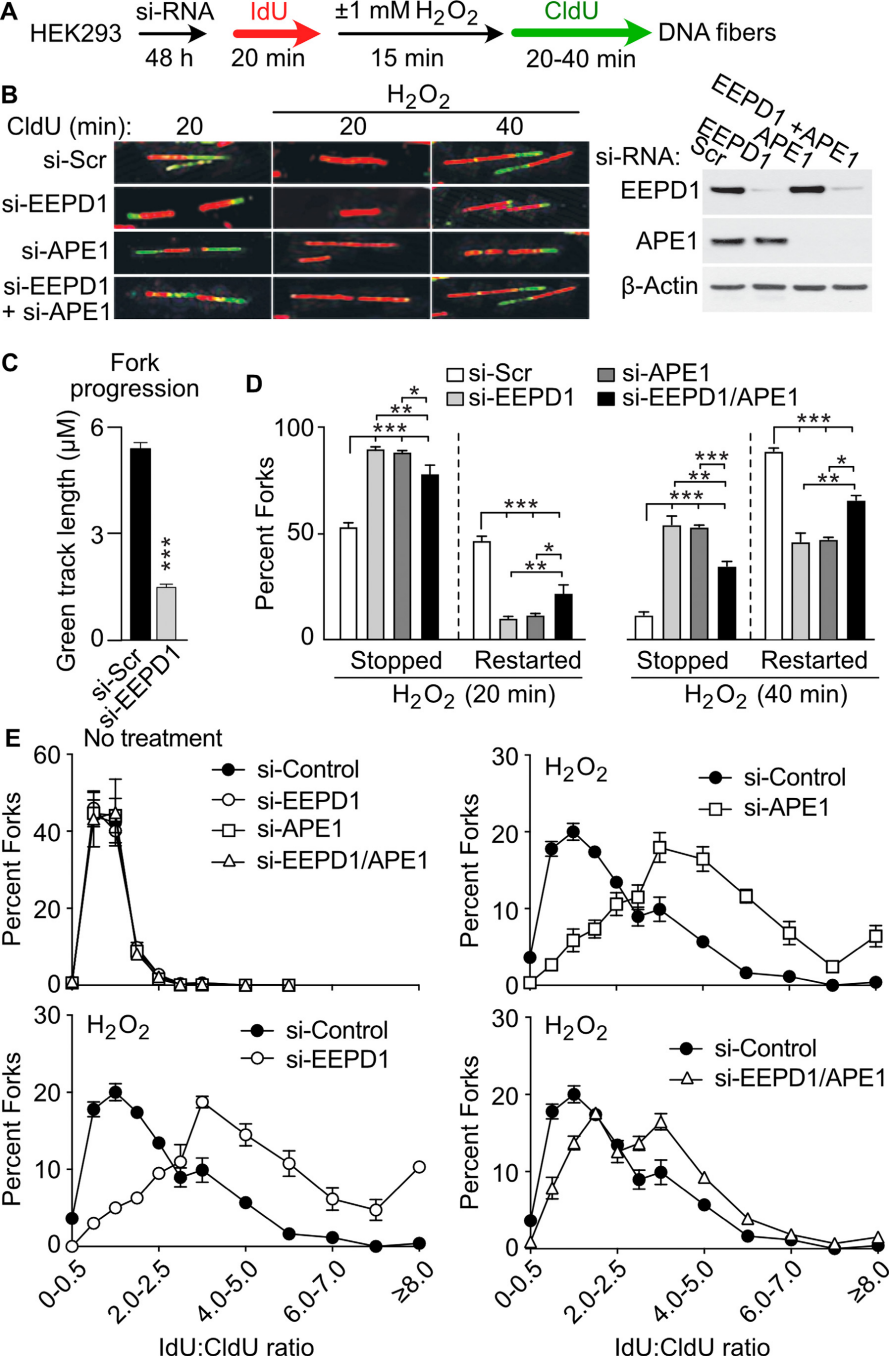
EEPD1 and APE1 promote restart of replication forks after oxidative stress in HEK293 cells. (**A**) A strategy used to analyze repair and restart of replication forks after oxidative stress in HEK293 cells with EEPD1 or APE1 depletion by DNA fiber analysis. (**B**) Representative images of DNA fibers from HEK293 cells depleted for EEPD1, APE1, or both and mock-treated or treated with H_2_O_2_. Western blots confirm single and double knockdowns. (**C**) EEPD1-depleted cells had decreased lengths of CldU-labeled tracks after oxidative stress, a measure of fork progression. **(D)** Fiber images were scored for stopped forks (IdU label only) and restarted forks (IdU and CIdU labels). Stopped forks increased significantly (and restarted forks correspondingly decreased) with depletion of EEPD1, APE1 or both proteins for fibers scored 20 or 40 min after incubation with CldU. Data are mean ± SE of three independent experiments. (**E**) IdU:CldU track length ratio distributions in control cells or cells depleted for EEPD1, APE1, or both proteins, with or without H_2_O_2_ treatment. Ratios expressed as mean ± SE of three independent experiments. In H_2_O_2_-treated cells, EEPD1 depletion results in relatively shorter CldU tracts, reflected as higher IdU:CldU ratio, indicating delayed fork restart and/or slower progression upon restart. Data are from three independent experiments, expressed as mean ± SE.

**Figure 3.**
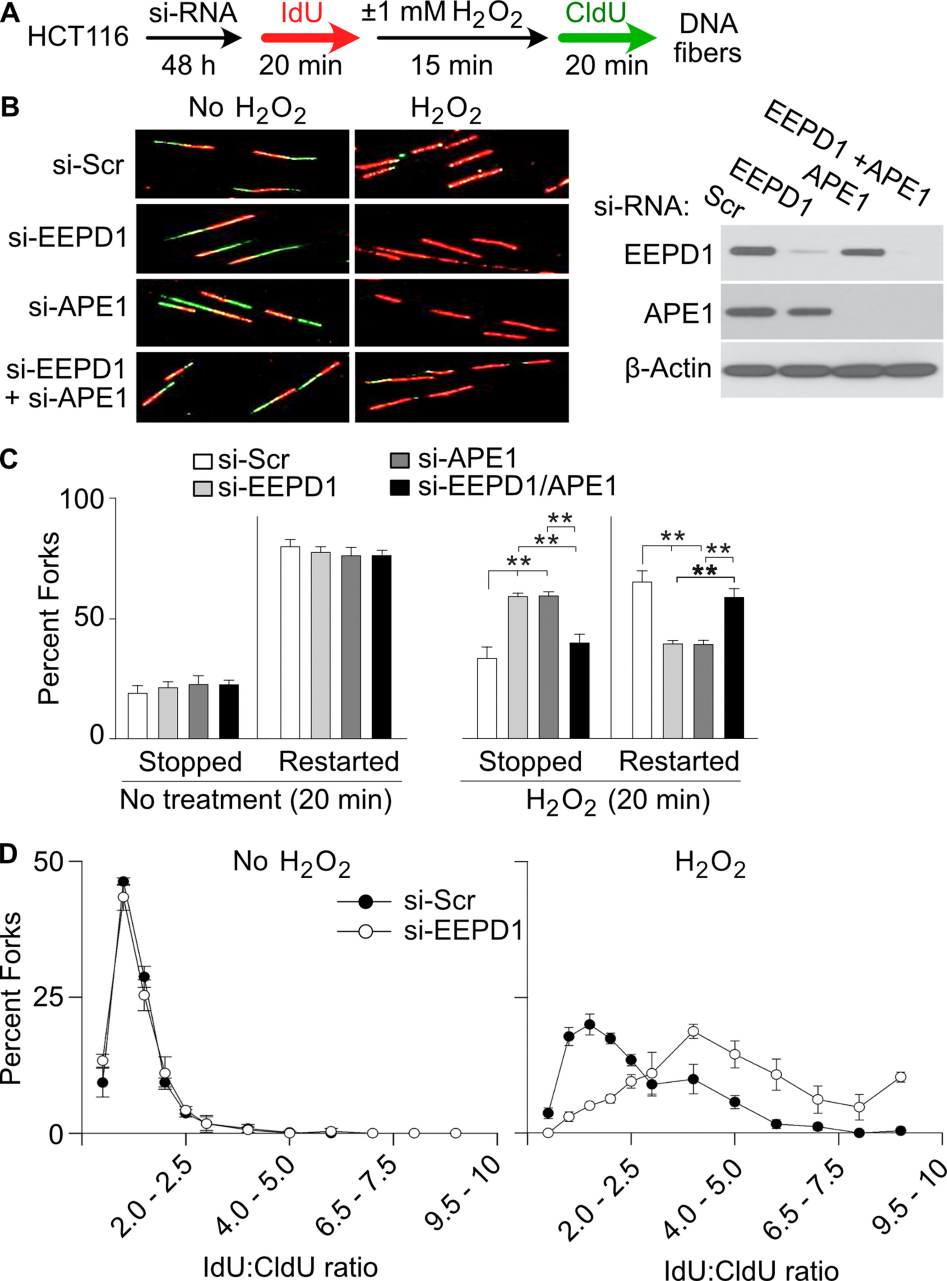
EEPD1 and APE1 promote repair and restart of replication forks after oxidative stress in HCT116 cells. (**A**) Fork restart strategy. (**B**) Images of DNA fibers captured with and without H_2_O_2_ treatment of HCT116 with scrambled siRNA (si-Scr) or siRNA to EEPD1, APE1, or both proteins. Western blots confirm single and double knockdowns. (**C**) DNA fibers were quantified and expressed as a percentage of restarted, stopped and newly initiated forks at indicated time points in mock treated or H_2_O_2_ treated cells. Data are the mean ± SE from three independent experiments. (**D**) IdU and CldU track length ratio distributions in control cells and cells depleted for EEPD1 with or without H_2_O_2_ treatment. In H_2_O_2_-treated cells, EEPD1 depletion results in relatively shorter CldU tracts, reflected as higher IdU:CldU ratio, indicating delayed fork restart and/or slower progression upon restart. Data are from three independent experiments, expressed as mean ± SE.

Since EEPD1 is important for replication fork repair and restart after nucleotide depletion (30,35,37–39), we asked whether EEPD1 played a role in the repair of oxidatively stressed replication forks. We used DNA fiber assays to test whether EEPD1 depletion in HEK293 or HCT116 cells would affect the restart of H_2_O_2_-stressed replication forks. We found that EEPD1 depletion significantly increased stalled forks and decreased restarted forks after oxidative stress in both 293 and HCT116 cell lines (Figures 2A–D, and 3A–C). In addition, replication forks that were able to restart after release from H_2_O_2_ had significantly shorter replication tracts in EEPD1-depleted cells (Figures 2E and 3D), implying that replication fork restart is delayed and/or restarted fork progression is hampered in EEPD1-depleted cells (30,35,37–39). EEPD1 defects do not affect fork progression in unstressed cells (30,39), suggesting that EEPD1 defects specifically inhibit fork repair and restart after oxidative stress. The effect of EEPD1 depletion on replication fork repair and restart after H_2_O_2_ stress was of similar magnitude as that caused by APE1 depletion (Figures 2D, E and 3C), implying that EEPD1 and APE1 have roles of similar magnitude but distinct functions in the repair and restart of oxidatively-damaged replication forks.

**Figure 4.**
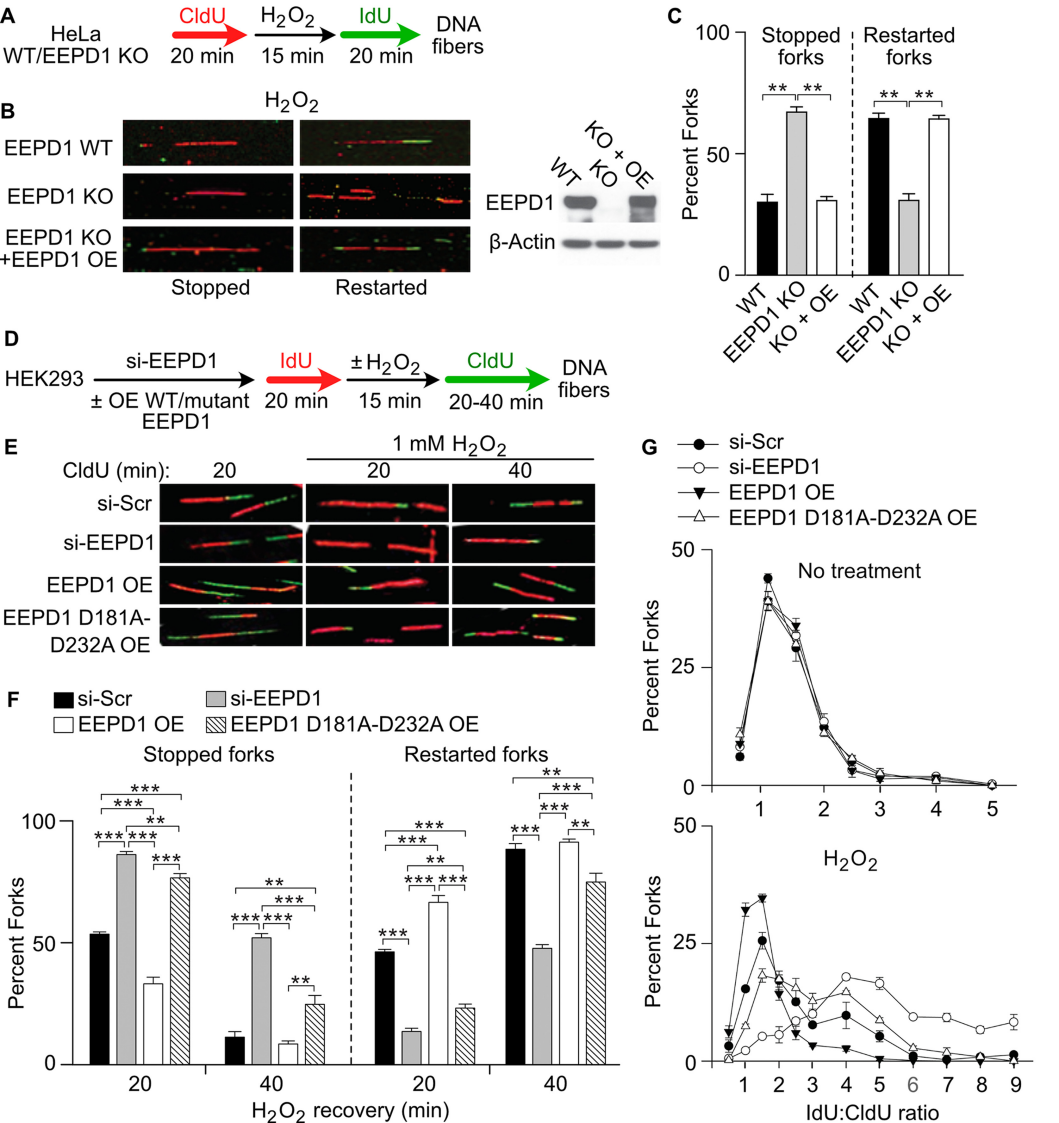
EEPD1 genetic deletion decreases repair and restart of replication forks after oxidative stress while its over-expression promotes it. (**A**) A strategy used to analyze replication fork restart after oxidative stress in HeLa cells with or without EEPD1 knock-out (KO) or overexpressed (OE) WT EEPD1. (**B**) Images of DNA fibers captured after H_2_O_2_ in HeLa cells with or without EEPD1 KO (IdU stained red, CldU stained green); western blot verifies EEPD1 KO and OE. EEPD1 KO has little effect on fiber outcomes in untreated cells (30). (**C**) Percentages of restarted, stopped and newly initiated forks after H_2_O_2_ treatment in HeLa wild-type or EEPD1 KO cells. Data are the mean ± SE from three independent experiments. (**D, E**) A strategy used to analyze fork restart in HEK293 cells with or without si-EEPD1 complemented with OE of wild-type or nuclease-dead D181A D232A EEPD1 and representative DNA fiber images. (**F**) Percentages of stopped or restarted forks after H_2_O_2_ treatment in HEK293 with or without si-EEPD1 and OE of wild-type or nuclease-dead EEPD1. Data are means ± SE from three independent experiments. (**G**) IdU:CldU track length ratio distributions in HEK293 control and EEPD1-depleted cells EEPD1 depletion/OE of WT or nuclease-dead EEPD1, and with or without H_2_O_2_ treatment. Ratios expressed as mean ± SE of three independent experiments. The shift toward lower IdU:CldU ratios with EEPD1 OE is further evidence that EEPD1 accelerates fork restart after H_2_O_2_.

We further observed that HeLa EEPD1 KO cells showed a similar decrease in H_2_O_2_-stressed replication fork repair and restart (Figure 4A–C), demonstrating that this effect is consistent across cell lines and with different methods of reducing EEPD1 expression. We further tested whether EEPD1 over-expression can facilitate replication fork repair. We overexpressed EEPD1 in Hela EEPD1 KO and HEK293 cells and analyzed DNA fibers after oxidative stress. Our data indicate that overexpression of EEPD1 enhanced the repair and restart of oxidatively-stressed replication forks (Figure 4D–F). This further indicates that the oxidative induction of EEPD1 (Supplementary Figure S2F) could enhance the repair of forks stressed by oxidative DNA damage.

We also tested whether the nuclease domain of EEPD1 played a role in the repair of the oxidatively stressed replication forks. We achieved this by overexpressing nuclease-defective EEPD1 D181A/D232A (35) in HEK293 cells followed by exposure to H_2_O_2_. We showed that the Flag-tagged EEPD1 constructs were able to localize in the nucleus similar to WT EEPD1 (30, Supplementary Figure S4), confirming the Protein Atlas and our previous data (30). Nuclease-defective EEPD1 D181A/D232A showed a defect in repair and restart 20 min after release from oxidative stress that was similar to EEPD1 depletion (Figure 4E–G). By 40 min after release, nuclease-defective EEPD1 showed a more moderate defect. These results suggest that the EEPD1 nuclease domain is important for maximum acceleration of fork repair and restart. In addition, overexpression of wild-type EEPD1 increased the length of restarted replication fork tracks (decreased IdU:CldU ratios), but this was not the case with the nuclease-defective mutant, further indicating that WT EEPD1 accelerates fork restart (Figure 4G). That complementation of EEPD1 restores the phenotype indicates that the phenotype is not an off-target effect of the siRNA. We further confirmed our findings by testing another 5′ endonuclease, Metnase. Metnase is another nuclease that promotes fork repair and restart after nucleotide depletion with hydroxyurea (39), yet Metnase did not affect the repair and restart of oxidatively-stressed replication forks (Supplementary Figure S5). Thus, based on these results we conclude that EEPD1 promotes cell survival after oxidative damage due to its function in the repair of oxidatively-damaged replication forks.

### EEPD1 protects replication forks after oxidative damage

**Figure 5.**
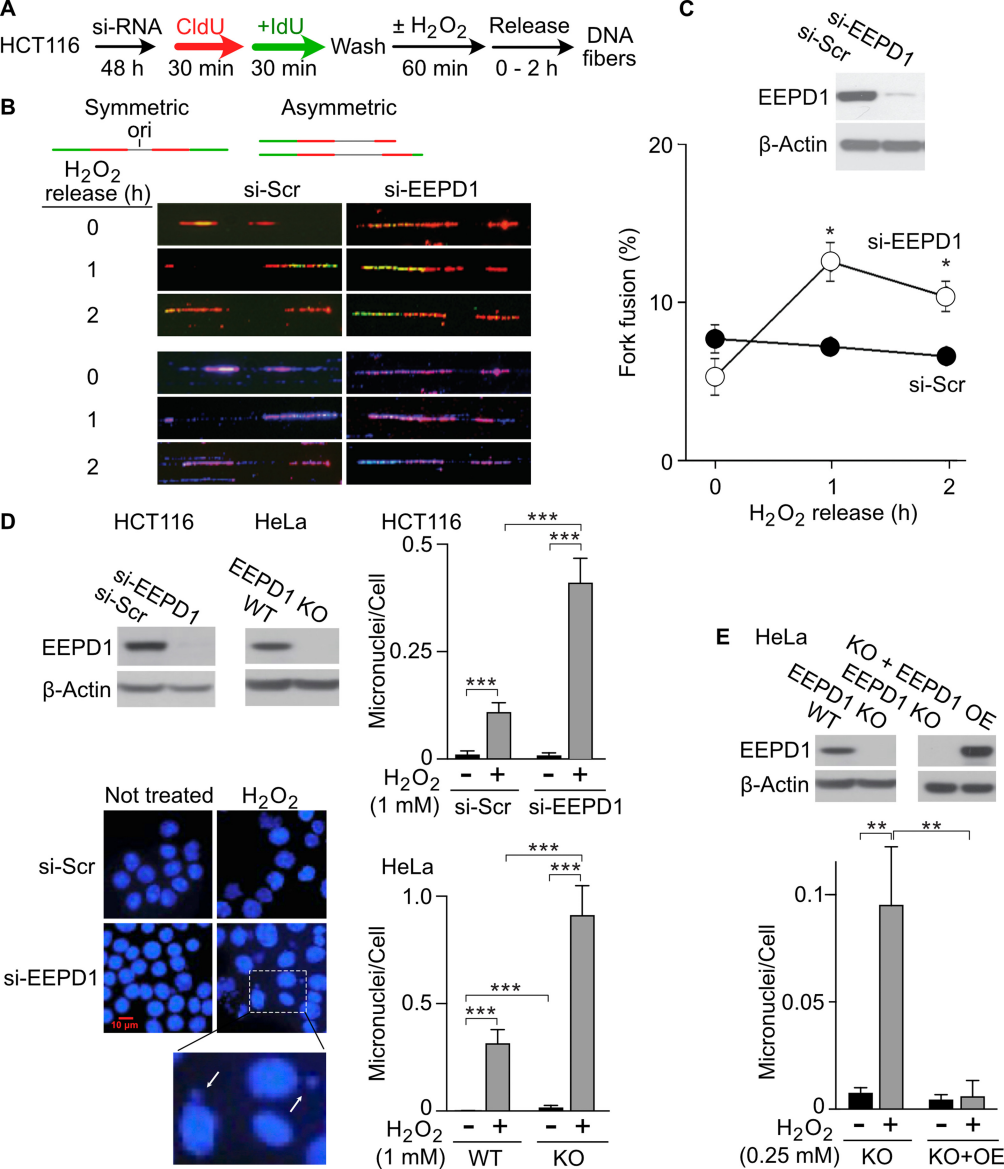
EEPD1 prevents fork fusion and micronuclei after oxidative stress. (**A**) Experimental strategy for the analysis of fork fusion after oxidative stress (1 mM H_2_O_2_) in HCT116 cells depleted of EEPD1. (**B**) Asymmetrically-labeled, asymmetrically-sized tracks on the same fiber after release from H_2_O_2_ represent a subset of unrepaired replication fork fusions. Schematics of symmetric and asymmetric fiber staining patterns shown above, representative images from >270 captured images in each condition of four independent experiments shown below. The top set of images shows IdU and CldU tracks, and the lower set shows DAPI staining. (**C**) Depletion of EEPD1 protein was confirmed by western blot, and fork fusion was quantitated in cells depleted of EEPD1 and exposed to H_2_O_2_. Images of DNA fibers were analyzed by ImageJ software. Data are the mean ± SE from the four independent experiments. (**D**) Retained chromosomes from fork fusions after oxidative stress were seen as micronuclei in HCT116 cells with siRNA depletion of EEPD1 and HeLa cells with WT EEPD1 or EEPD1 KO. Western blots confirm EEPD1 knockdown and knockout. Representative images of DAPI-stained HCT116 show nuclei and micronuclei (inset, arrows). Micronuclei quantitation is plotted as mean ± SE after 1 h treatments with indicated H_2_O_2_ concentrations. (**E**) Complementation of EEPD1 knockout with overexpressed (OE) WT EEPD1 suppresses oxidative stress-induced micronuclei; western blots confirm knockout and complementation.

There are two major adverse outcomes for unrepaired replication forks, fork degradation and fork fusion (9–13,53–56). If repair fails, free DNA ends at reversed or cleaved forks can undergo nucleolytic degradation or can be aberrantly ligated with another free DNA end from a distinct fork to generate chromosome translocations or other rearrangements (9,30,35,37–39). Adjacent forks on the same fiber with asymmetric sizes and asymmetric CIdU:IdU-labeled tracks (Figure 5B, upper panel) represent a subset of replication fork fusion events. Using this method, we discovered that EEPD1-depleted HCT116 cells had 1.8-fold more asymmetrically sized and asymmetrically-labeled forks on the same fiber after H_2_O_2_ exposure than control cells (Figure 5A–C). When a fork fusion results in a chromosomal translocation that deletes the centromere, that chromosome will be retained after mitosis. Retained chromosomes generate micronuclei, a sign of genomic instability (12,40). We measured micronuclei formed in siRNA-depleted EEPD1 in HCT116 cells or EEPD1 KO HeLa cells exposed to oxidative stress. We found that micronuclei were significantly increased in these EEPD1-defective cells after oxidative stress (Figure 5D). To further validate our findings, we overexpressed EEPD1 in HeLa EEPD1 KO cells and again measured micronuclei after oxidative stress and found that EEPD1 complementation of HeLa KO cells rescued the mitotic chromosomal abnormalities (Figure 5E). However, it is recognized that micronuclei can arise from multiple sources, not just retained chromosomes that have lost their centromere due to acentric translocations such as suggested here (12,40).

The other adverse outcome of free DNA ends at unrepaired replication forks is fork degradation. We assessed the degradation of unrepaired forks by measuring the ratio of the length of the second label (newest synthesized DNA) to the first label on individual fibers (53–56). We found that there was a statistically significant albeit small increase in fork degradation (1.4-fold) in EEPD1-depleted H_2_O_2_-treated HCT116 cells (Supplementary Figure S6). EEPD1 may prevent fork degradation by promoting HR-mediated fork restart, analogous to the dual role of BRCA2 in HR and fork protection (30,35,38,54). Supplementary Figure S6 shows that smaller IdU:CldU ratios are most prominent for the shortest IdU tracks (IdU:CldU ratios between 0 and 0.5), where short IdU tracts (lowest IdU:CldU ratio) increase from 14% to nearly 20% of measured tracks, a statistically significant increase (*P* < 0.05), implying that the lowest IdU:CldU ratio is the most sensitive measure of fork degradation.

These results suggest that EEPD1 prevents adverse genomic outcomes of oxidatively-stressed replication forks, preventing fusion and to a lesser extent degradation of unrepaired forks. This is likely due to EEPD1 enhancing the repair of the oxidatively-stressed fork before it can undergo fusion or degradation, thereby promoting genome stability and cell survival in response to oxidative DNA damage.

### Recruitment of EEPD1 to the oxidatively-stressed replication forks

**Figure 6.**
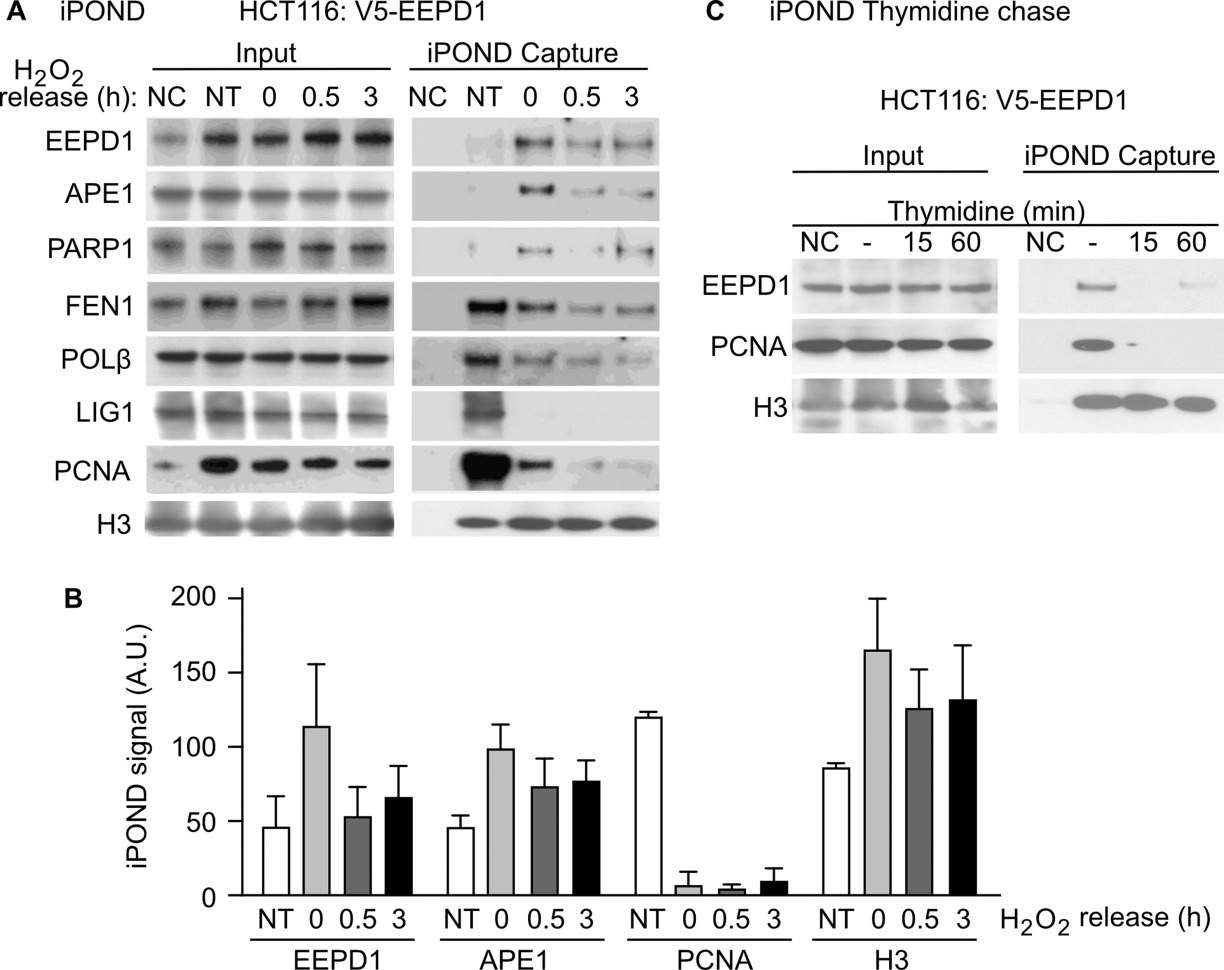
EEPD1 and APE1 are recruited to replication forks after oxidative stress. (**A**) HCT116 cells expressing V5-tagged EEPD1 were subjected to iPOND analysis after treatment with 0.5 mM H_2_O_2_ and followed by thymidine chase. IPOND analysis of EEPD1 and BER proteins at replication forks after oxidative stress. IPOND input and capture samples were probed 0–3 h after release from 0.5 mM H_2_O_2_ for EEPD1, APE1, POLβ, FEN1, LIG1, PARP1, PCNA and H3 (PCNA and H3 served as loading and stressed fork response controls). NT- not treated; NC- no click control. Representative images from three independent experiments are shown. (**B**) Densitometric analysis of iPOND capture signals, plotted as averages (±SEM) for three determinations. (**C**) Elimination of EEPD1 iPOND signal after brief thymidine chase after release from stress demonstrates EEPD1 presence near replication forks in response to oxidative stress.

If EEPD1 initiates repair and restart of oxidatively-stressed replication forks, then it is predicted to be present at forks in response to oxidative stress. We indeed found that while there was a low level of EEPD1 present at untreated replication forks, perhaps because cells are cultured in a relatively high oxygen atmosphere, its recruitment to forks was significantly induced by H_2_O_2_ as measured by iPOND assays (Figure 6A, B) (48,49). Removing the H_2_O_2_ and chasing the cells with thymidine led to decreased EEPD1 at the replication fork, indicating that EEPD1 recruitment to forks was indeed induced by oxidative stress (Figure 6C).

We next used iPOND assays to determine whether proteins that are constitutive members of the BER complex are present at stressed or unstressed replication forks by using iPOND assay, including APE1, PARP1, FEN1, POL β, DNA ligase I (LIG1), and PCNA. Our results indicate that PCNA, FEN1, POL β and LIG1 are present at unstressed replication forks, while EEPD1, APE1, and PARP1 levels are increased at replication forks after oxidative stress (Figure 6A, B). LIG1 and PCNA quickly dissociate from the replication fork after H_2_O_2_ treatment, likely due to the discontinuation of replication. Protein levels of EEPD1 and PARP1 increased at the replication fork after H_2_O_2_ treatment while FEN1 and POL β levels receded after oxidative stress (Figure 6A). When cells were released from oxidative stress, levels of both APE1 and EEPD1 at the replication fork receded in a time-dependent manner. However, EEPD1 appears to persist at higher relative levels for a longer period than APE1. It was noted that reduced levels of APE1, PARP1, FEN1 and POL β proteins were present at oxidatively-stressed replication forks 3 h after the H_2_O_2_ release. The equal level of histone H3 detected on isolated chromatin shows an equivalent amount of EdU-labeled DNA was purified in each sample. The input samples (Figure 6A, left panel) indicate the level of these proteins. These results suggest that EEPD1 is recruited to oxidatively stressed replication forks and persists there longer than APE1. We further validated by thymidine chase that EEPD1 is independently recruited to the replication fork (Figure 6C, right panel).

### EEPD1 resolves oxidative DNA damage

**Figure 7.**
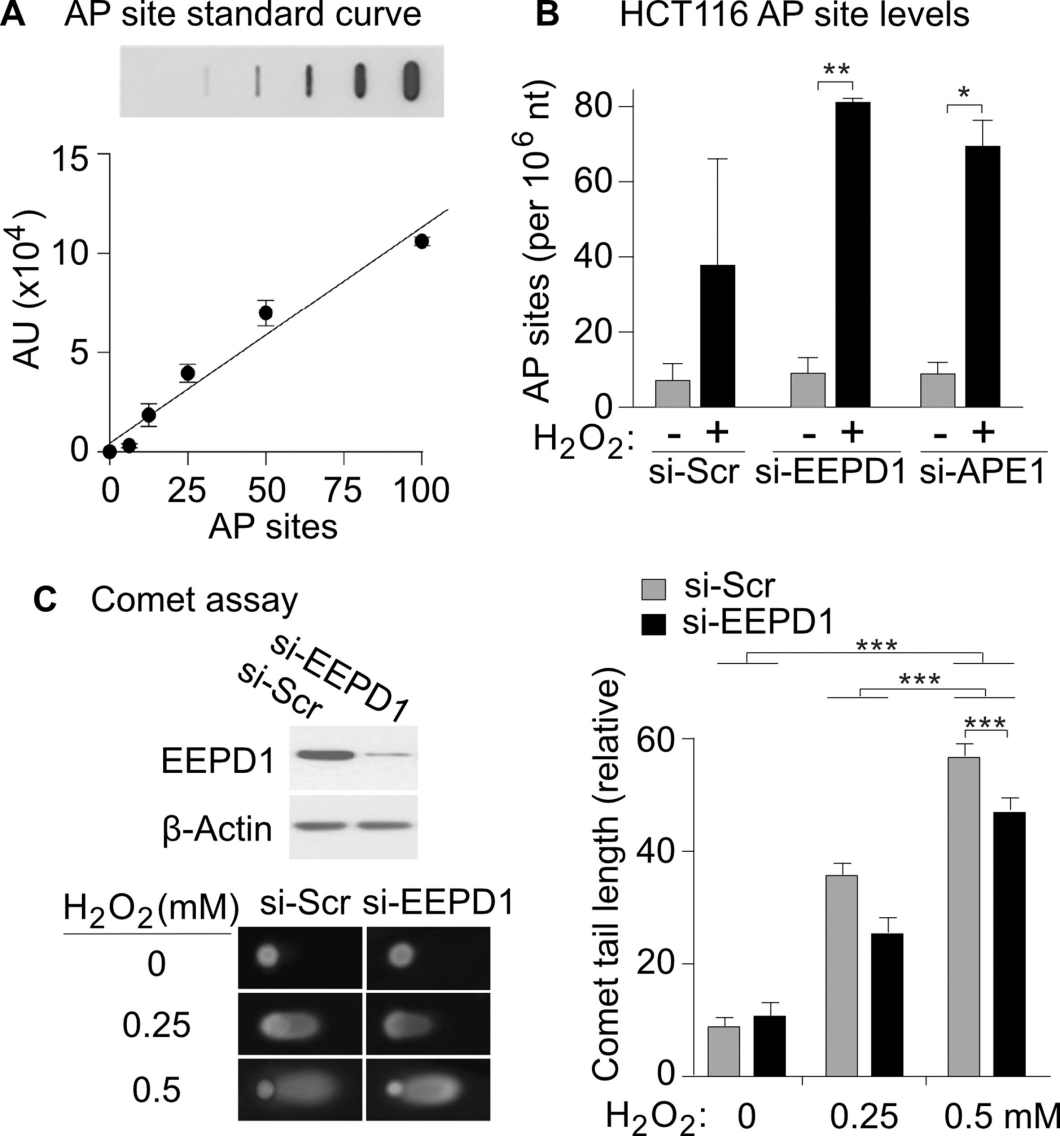
EEPD1 deficiency increases replication fork oxidative damage. (**A**) Standard curve for AP site quantitation from slot blot. (**B**) AP site detection in slot blots of control, si-EEPD1, or si-APE1 depleted HCT116 cell DNA, with or without H_2_O_2_ treatment; quantitation of AP sites from two independent slot blot experiments. (**C**) EEPD1 contributes to genomic single-strand cleavage during H_2_O_2_ stress as determined by alkaline comet assays. Shown are western blots confirming EEPD1 depletion, representative comet tail images with or without H_2_O_2_ treatment, and quantitation of comet tail lengths with mean ± SE from three independent experiments.

Genomic AP sites are repair intermediates formed by glycosylase removal of damaged bases. EEPD1 endonuclease activity could resolve these sites by 5′ single-strand cleavage to initiate BER, similar to APE1. Consistent with this hypothesis, we directly measured AP sites in genomic DNA before and after oxidative stress (41,57). EEPD1 depletion increased the number of AP sites in untreated cells, whose oxidative damage results from cellular metabolism and/or from cell culture in 20% atmospheric oxygen, and AP sites further increased with EEPD1 or APE1 depletion upon exposure to H_2_O_2_ (Figure 7A, B). If EEPD1 plays a role in resolving genomic oxidative DNA damage via its 5′ AP endonuclease activity, its depletion should generate fewer single-strand nicks after H_2_O_2_. We further observed that EEPD1 depletion resulted in decreased genomic single-strand cleavage during H_2_O_2_ stress, as determined by alkaline comet assays (Figure 7C). This was also reflected in suppressed 53BP1 and γH2AX focus formation after oxidative stress in cells with EEPD1 depletion (Supplementary Figure S7). It is recognized that alkaline conditions in Comet assays can cleave abasic sites. However, if EEPD1 plays a role in resolving abasic sites after oxidative DNA damage via its 5′ AP endonuclease activity, its depletion should generate fewer single-strand nicks after H_2_O_2_, which is what we observed. Thus, we conclude that the role of EEPD1 in cleaving abasic sites modestly exceeds that of abasic site cleavage by the alkaline conditions. Without the alkaline cleavage of residual abasic sites, this difference would have been even greater.

### EEPD1 binds to DNA with an AP site

**Figure 8.**
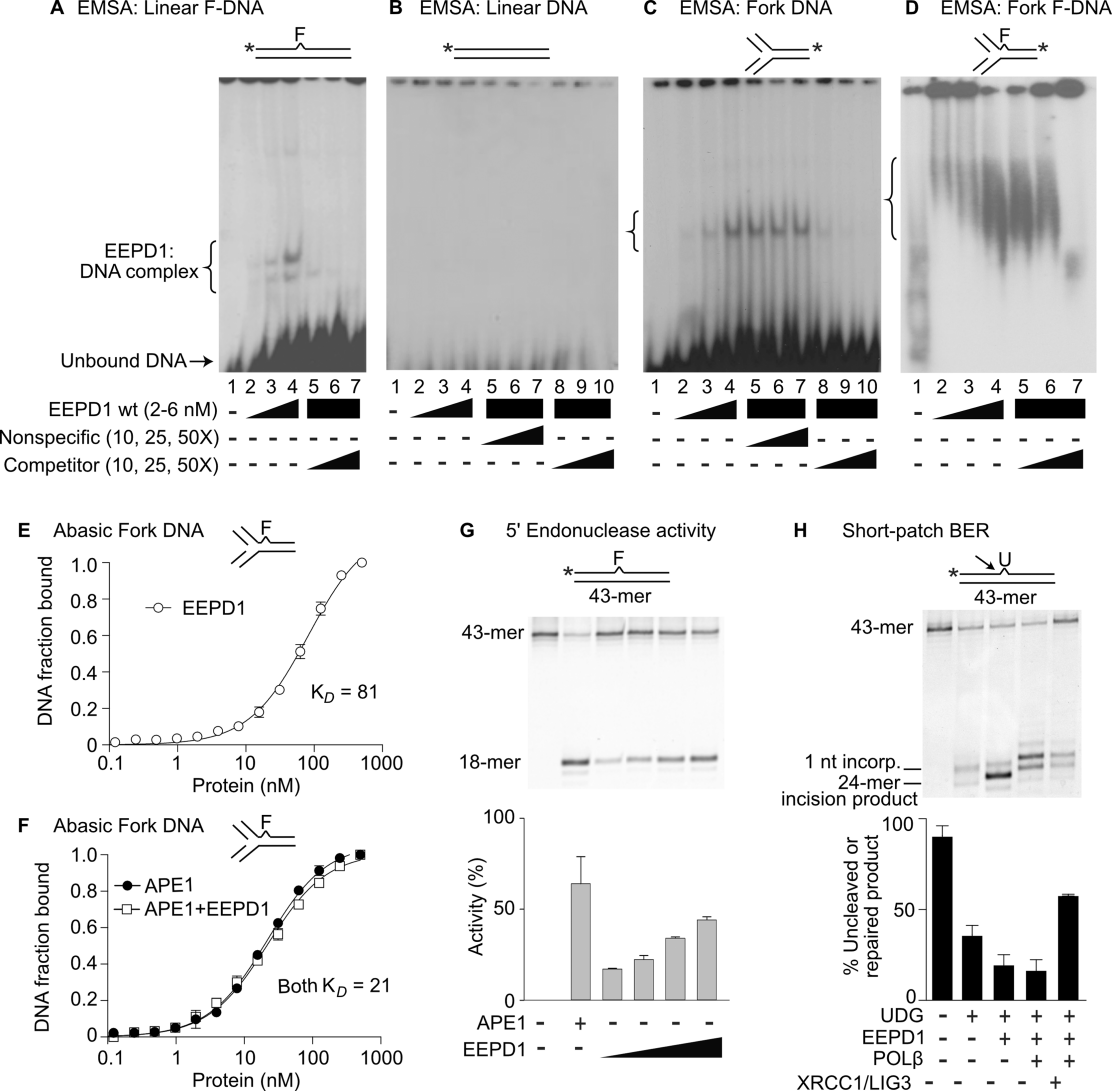
EEPD1 binds to and cleaves abasic sites in linear and fork DNA and catalyzes SP-BER. (**A**, **B**) EEPD1 (2–6 nM) binds to linear F-DNA but not undamaged linear DNA in EMSA assays. Representative images from three independent experiments are shown. (**C**, **D**) EEPD1 binds to fork DNA with or without an abasic site. (**E, F**) Estimation of dissociation constant (K*_D_*) and binding affinity of EEPD1 and/or APE1 on a abasic fork DNA. Data are mean ± SE of three independent experiments. (**G**) Dose-dependent 5′ endonuclease cleavage by EEPD1 at a THF abasic site. Respective activities are quantified by densitometry in the graphs below the image. Plots are mean ± SE of three independent experiments using distinct protein preparations. (**H**) SP-BER mediated by EEPD1 in reactions with a uracil DNA substrate and BER components. Images are representative of three independent experiments using distinct protein preparations. The densitometric quantitation of cleaved (unrepaired) products at each step are below the image. The far-right lane of the image and graph represents the completely repaired product.

Using an electrophoretic mobility shift assay (EMSA), we tested whether full-length WT recombinant human EEPD1 (Supplementary Figure S2A, B) binds to duplex DNA oligomers with or without a 3-hydroxy-2-hydroxymethyltetrahydrofuran (THF) site that mimics an AP lesion (58). We found that EEPD1 bound more efficiently to duplex DNA with THF than to unmodified duplex DNA (Figure 8A, B). EEPD1 also binds to an oligomer fork structure (Figure 8 C, D). EEPD1 is a structure-selective endonuclease that cleaves fork structures (30,35). In the present study, we found that EEPD1 binds to fork structures, and this binding is enhanced at fork structures with a THF site (Figure 8C, D). It is entirely possible that EEPD1 binds to an AP site in the genome because it resembles the SS bend in a stalled fork structure, and not because that nucleotide has lost its base. In any case, the end result would still be AP site 5′ SS nicking that could serve as a template for BER or even 5′ end resection to initiate HR and prevent replication fork fusion and subsequent aneuploidy (9–13,30,35,59).

We previously reported that APE1 preferentially binds to THF-containing duplex oligomers in EMSA (41,57). We next measured the relative affinity of EEPD1 compared to the full-length APE1 binding to a double-stranded replication fork structure with a THF site. Using fluorescence polarization, we found that EEPD1 bound to fork DNA with a single THF site with a *K*_D_ of 81 nM, while APE1 bound to the same DNA structure with a *K*_D_ of 21 nM demonstrating that APE1 binds 3.8-fold more efficiently to the THF fork DNA than EEPD1 (Figure 8E, F). The lower affinity of EEPD1 for AP site DNA compared to APE1 and the higher relative APE1 expression suggests that EEPD1 may serve to back up APE1 in the repair of oxidative damage.

### EEPD1 can mediate BER

To identify a mechanism by which EEPD1 improves the repair and restart of oxidatively-damaged replication forks and thereby enhances cell survival, we assessed whether it could play a direct role in BER. Since EEPD1 binds to DNA structures containing a THF abasic site (Figure 8A, D), we next tested whether EEPD1 can complete SP-BER reconstituted in an *in vitro* assay system in absence of APE1 (14,15,41). Similar to APE1, we found that EEPD1 incises duplex DNA 5′ to an AP site (Figure 8G). Reconstituting SP-BER in an *in vitro* assay (15,41), we found that EEPD1 can replace APE1 and promote the completion of SP-BER. This reconstituted assay system used a DNA substrate with single uracil, with uracil DNA glycosylase (UDG) treatment creating an AP site, and POL β and XRCC1/LIG3 added to complete the BER reaction (Figure 8H). The marked differences in APE1 and EEPD1′s specific activities (∼100-fold) in this assay may reflect actual enzymatic dfferences or it could derive from challenges with post-translational modifications or other difficulties with protein isolation, given that EEPD1 is far more difficult to purify, or it could be due to the differences in binding efficiencies noted above, but regardless, our results are consistent with EEPD1 serving in a backup role to APE1 in BER.

## DISCUSSION

Repair and restart of the oxidatively-stressed replication fork is imperfectly understood. Published data suggest that NEIL1/3 can initiate BER in front of replication forks to prevent stalling (17–19,60). However, when there are high levels of oxidative stress, most replication forks will still stall, implying that the NEIL1/3 ‘cowcatcher’ function can be overwhelmed. Since even small delays in replication result in a high incidence of cell death (30), we hypothesized that another pathway exists to repair and restart oxidatively-stressed replication forks.

EEPD1 enhances stressed replication fork repair by cleaving the lagging parental strand flap of a double-stranded replication fork, thereby promoting EXO1 loading to enhance 5′ end resection and initate HR (30,35,37–39). To further define the role of EEPD1 in DNA repair, we surveyed cell survival after EEPD1 depletion in the presence of various DNA-damaging agents. We found that EEPD1 was important for survival after exposure to MMS and H_2_O_2_, agents that cause damage repaired by BER (Figure 1). Furthermore, we found that EEPD1 was important in resolving oxidative damage at replication forks (Figures 2–4). Based upon these observations, we explored the mechanism of this phenomenon and made the unexpected discovery that EEPD1 cleaves the phosphodiester bond immediately 5′ to the abasic site, similar to APE1 (Figure 8G). We also found that EEPD1 can mediate SP-BER in a reconstituted reaction (Figure 8H). However, EEPD1 has 4-fold lower avidity for DNA with an AP site than APE1 (Figure 8E) and >100-fold less *in vitro* 5′ AP endonuclease activity than APE1 (Figure 8G). In addition, APE1 is ∼60 times more abundant than EEPD1 in many cell lines based on its transcript levels, although this does not necessarily correlate with protein levels. However, it is significantly more difficult to isolate EEPD1 than APE1 protein and this may account for some of the difference in activity. Nonetheless, the results here suggest that the canonical APE1-mediated BER pathway predominates in most situations and EEPD1 mediates a back-up pathway.

There are three potential, non-exclusive reasons why EEPD1-mediated BER might be important for oxidative DNA damage repair. First, there are several types of DNA lesions that cannot be processed by APE1, suggesting the existence of alternate BER pathways that would employ other nucleases (28,29,61–63). For example, APE1 poorly cleaves branched abasic sites such as 2-(aminobutyl)-1,2-propanediol residues, abasic lesions close to the free end of a duplex, and abasic sites with a base-pair mismatch on either side (62). In addition, APE1 cannot cleave abasic sites in certain DNA structures, such as duplex DNA loops that mimic telomere structures (28). APE1 also poorly cleaves certain forms of clustered abasic sites, especially where there are lesions on the strand opposite of the target abasic site (63). Such lesions are induced by ionizing radiation, especially high linear energy transfer radiation (63). Consistent with the possibility that EEPD1 facilitates the repair of clustered abasic sites, we reported that EEPD1 contributes to cell survival after ionizing radiation (30). Second, it is possible that EEPD1 serves as a backup BER pathway when there is oxidative damage saturates the capacity of canonical BER components. The finding that EEPD1 depletion decreases cell survival at higher concentrations of H_2_O_2_ and MMS supports this possibility. Third, since both APE1 and FEN1 have many other essential functions, EEPD1 in its backup role could permit their utilization elsewhere in an oxidative crisis. The vast majority of oxidative DNA lesions do not occur at replication forks, but replication fork damage is likely the most deleterious type of oxidative damage and is thus the most probable to engender selective evolutionary pressure for such a backup pathway (1–4,9–13). EEPD1 persists at oxidatively-stressed replication forks longer than APE1, implying that there may be oxidative lesions that EEPD1 is required to repair, such as those blocking replication forks (Figure 6).

EEPD1 depletion arrests embryonic CNS development in zebrafish and causes severe DNA damage and genomic instability, indicating that EEPD1 is required during the rapid cell division of embryogenesis (37). Neoplasia can also demonstrate high replication rates and can have high oxidative damage rates; thus, some may require a backup pathway for the repair of oxidatively damaged replication forks (64,65). A fraction of tumors express APE1 at low or undetectable levels, yet they retain their proliferative potential, leading to tumorigenesis (20–23). There are at least two reports of healthy APE1 knock-out cell lines (22,23,41). In addition, some aging organs, such as the liver or CNS lose expression of APE1 without any immediate overt biological effect (24–27,66). In these situations, EEPD1 may assume a more critical role in maintaining genome stability.

Interestingly, despite EEPD1 depletion most oxidatively-stressed replication forks are ultimately repaired and restarted, yet EEPD1 depletion is still lethal to a fraction of cells undergoing oxidative stress (Figures 1–4). This implies that cells do not tolerate delays in replication fork repair. Indeed, replication forks will bypass DNA lesions and cells will repair the lesions later, rather than delaying replication to complete lesion repair (30,67,68). As mentioned above, even short delays of a few minutes may trigger apoptosis in cells, because of the dire consequences of stalled and unrepaired replication forks, including fork fusion and fork degradation (30,53–56,67,68), both of which were significantly increased in EEPD1-depleted cells after oxidative DNA damage (Figure 5A–C and Supplementary Figure S6).

We previously reported that EEPD1 promotes 5′ DNA end resection in a BRCA1-independent manner by cleavage of stalled forks and recruitment of EXO1 to initiate 5′ end resection (30,35,37–39). Since BRCA1 is important for initiating end resection in normal cells, BRCA1-mutant cancer cells use EEPD1 to cleave stressed replication forks and initiate end resection during RAD52-mediated HR repair of stressed replication forks (38). However, because normal cells have intact BRCA1, this alone would not explain the evolutionary selection of EEPD1 for stressed replication fork repair. Based on the results presented here, we postulate that the repair of oxidatively damaged replication forks represents the primary evolutionary function of EEPD1. It is possible that increased replication stress associated with genome expansion created selective pressure to evolve EEPD1 to enhance the repair of stressed replication forks.

**Figure 9.**
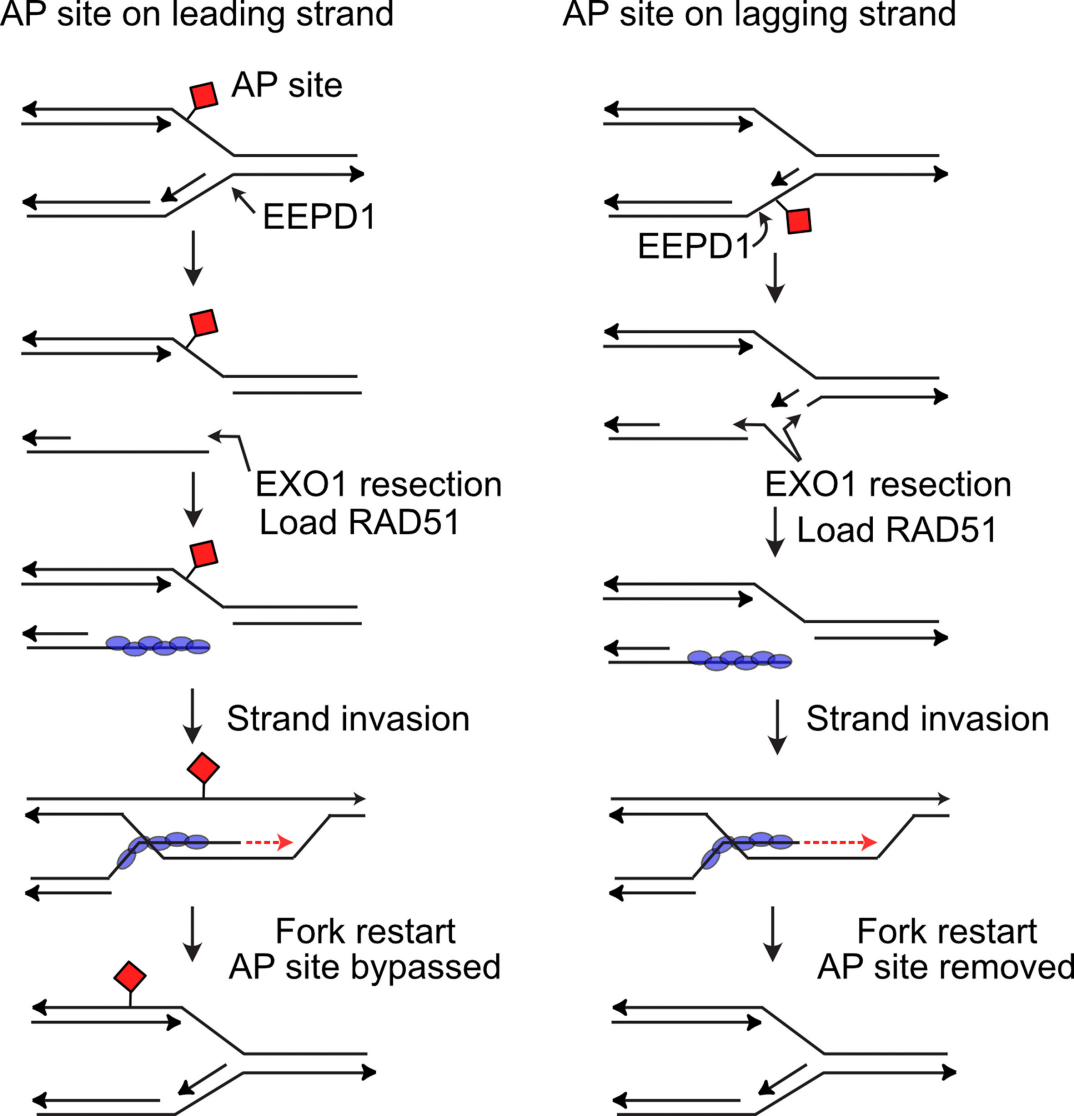
Models of EEPD1 repair and restart of oxidatively-damaged replication forks. Oxidatively damaged nucleotides are processed by a glycosylase to generate an AP sites on leading or lagging template strands, which can block replicative polymerases. Left: EEPD1 is recruited to AP site-stalled fork and cleaves the lagging strand template, as we demonstrated previously for HU-stalled replication forks (30,35). We found that this cleavage enhances loading of EXO1 for 5′ end resection, permitting HR-mediated fork restart bypassing the AP site blocking leading strand (35). Right: EEPD1 cleaves the lagging parental strand 5′ of the AP site, promoting EXO1 5′ end resection, which removes the AP site (right). In either case, the 5′ end resected single-stranded lagging strand template is coated with RAD51, allowing invasion of the duplex DNA to restart the fork.

It is tempting to speculate that the roles of EEPD1 may be seamlessly integrated, where EEPD1 cleavage of the oxidatively-stalled replication fork leads to HR repair and restart (30,35, see models in Figure 9). In these models, EEPD1′s abasic endonuclease activity at an oxidatively-stressed replication fork could lead to 5′ end resection for HR repair and restart of the damaged fork instead of completion of BER. Nicks at abasic sites could lead to loading of the EXO1 complex for end resection as we described (35). In this scenario, the initial steps of the EEPD1-mediated alternative BER pathway could also serve as an initial step towards HR. This would be a more efficient path for oxidatively-stressed replication fork restart as compared to waiting for completion of BER and then initiating HR-dependent fork restart.

This model is consistent with BRCA1-mutant cancer cells subverting EEPD1 for stressed fork repair to initiate a RAD52-mediated fork restart pathway as mentioned above (38). BRCA1-mutant cancers have markedly increased oxidative stress, which would require robust BER pathways to repair damaged DNA and maintain cell viability (69,70). Thus, our prior analysis of EEPD1 in BRCA1-mutant cells (38) and the present results suggest that EEPD1 plays an important role in permitting BRCA1-mutant cancers to survive despite increased oxidative DNA damage including replication fork damage.

Interestingly, depleting EEPD1 in cells where APE1 is also lacking led to increased replication fork restart after oxidative stress. This is consistent with our previous report that EEPD1 mediates a toxic repair intermediate when RAD52-mediated replication fork repair is not completed in BRCA1-deficient cells (38). The same could be occurring here, where EEPD1 generates a toxic repair intermediate when APE1 depletion prevents the completion of oxidatively-stressed replication fork repair. This also implies that EEPD1 and APE have distinct functions at the oxidatively-stressed replication fork, alhough both functions could be epistatically related.

Oxidative stress at replication forks is a common problem in many malignancies, not just BRCA1-mutant cancers, as cancer cell metabolism generates oxygen free radicals to a higher extent than normal cells. As mentioned, neoplastic cells also display forced DNA replication by virtue of their highly proliferative state, increasing the risk of replication forks encountering oxidative damage (64,65). This may explain why mutations in EEPD1 are rare in cancers and the observation that EEPD1 is expressed at higher levels in most tumor types than in normal cells (Supplementary Figure S2E) (30). Our results suggest that for those cancers that are more susceptible to oxidative damage, such as glioblastomas targeting EEPD1 might present a novel therapeutic target.

## DATA AVAILABILITY

All relevant data are within the paper and its Supporting Information file. The relevant primary data is available upon request and a signed materials transfer agreement.

## Supplementary Material

zcac044_Supplemental_File
