# Supplementary material for: EEPD1 promotes repair of oxidatively-stressed replication forks
Source: NAR Cancer. 2023 Jan 18;5(1):zcac044. doi: 10.1093/narcan/zcac044 (PMC9846428; doi:10.1093/narcan/zcac044)
Supplement: zcac044_Supplemental_File [file zcac044_supplemental_file.docx]

**Supplementary Information NARC-2020-090**

**EEPD1 Promotes Repair of Oxidatively-Stressed Replication Forks**

Aruna S. Jaiswal, Hyun-Suk Kim, Orlando D. Schärer, Neelam Sharma, Elizabeth A. Williamson, Gayathri Srinivasan, Linda Phillips, Kimi Kong, Shailee Arya, Anurag Misra, Arijit Dutta, Yogesh Gupta, Christi A. Walter, Sandeep Burma, Satya Narayan_,_ Patrick Sung, Jac A. Nickoloff, and Robert Hromas

**Supplementary Figures S1-S7**

**Supplementary Tables S1, S2**


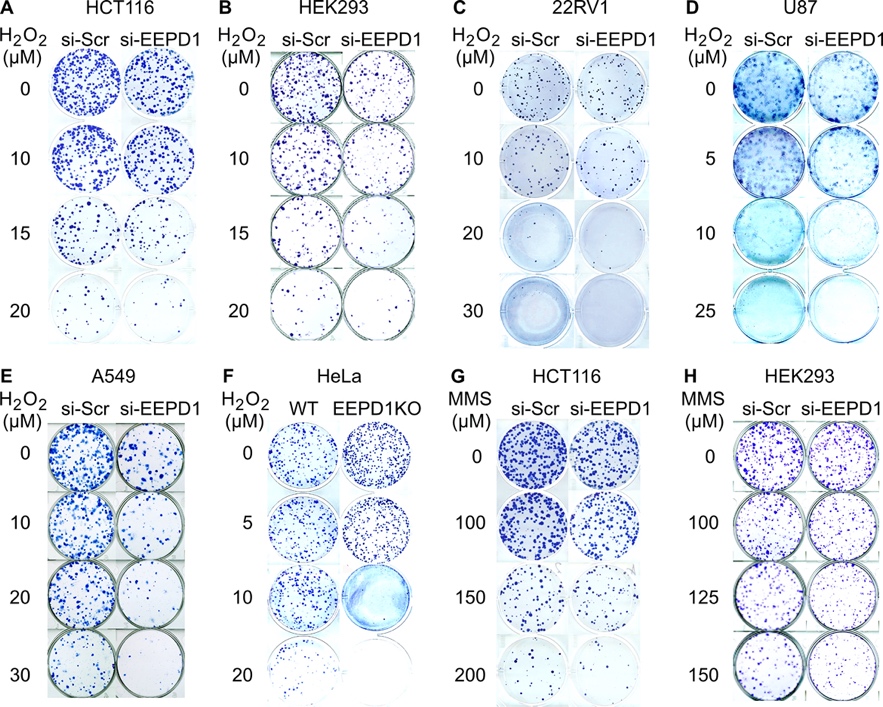


**Figure S1.** EEPD1 promotes cell survival after oxidative and alkylating DNA damage. (**A-E**) Cells transfected with siRNA for 24 h were treated for 24 h with 0-30 μM H_2_O_2_, and colonies were imaged after 14 d incubation. (**F**) HeLa WT and EEPD1 KO treated with H_2_O_2_. (**G-H**) Cells transfected with siRNA as above treated with MMS.


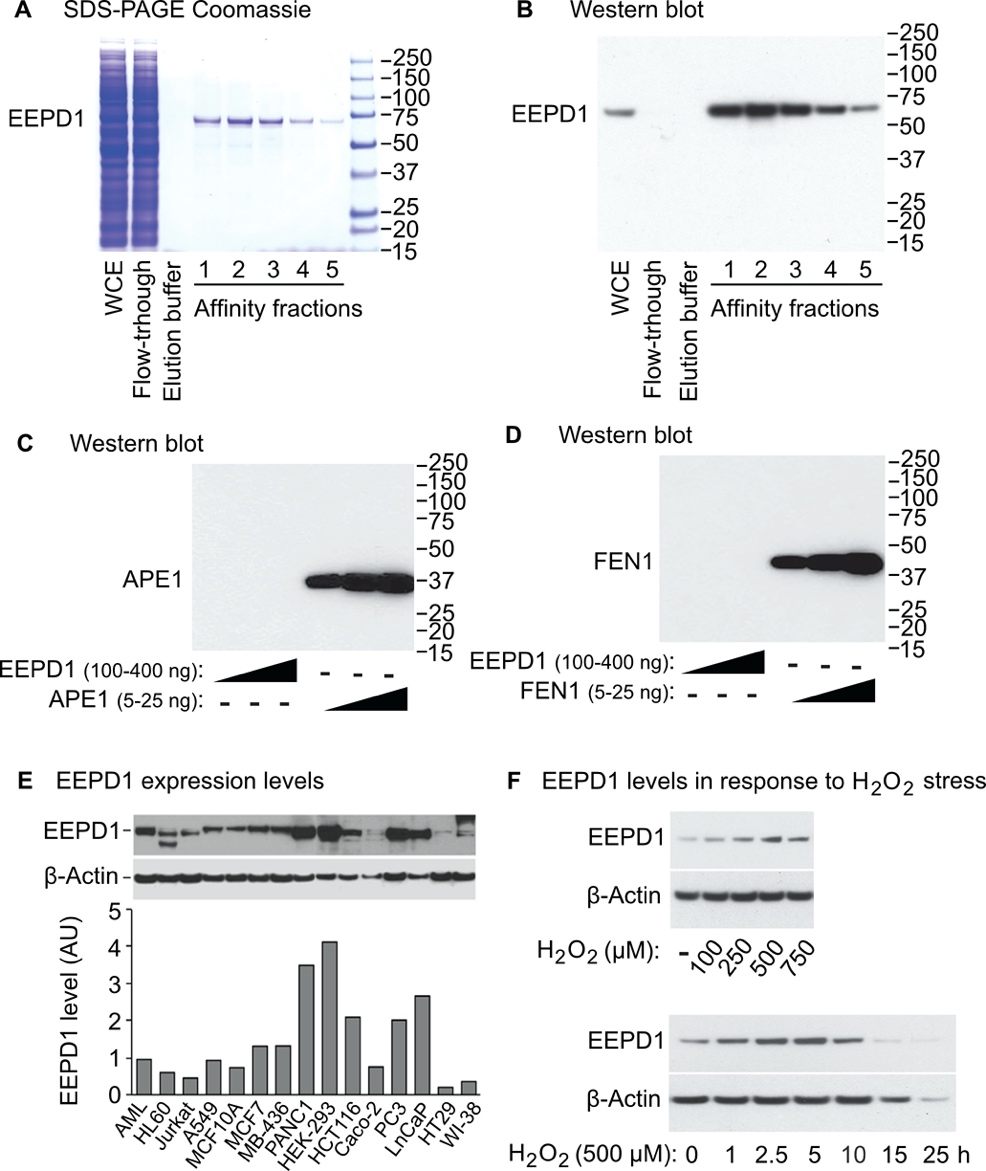


**Figure S2.** Purification and characterization of recombinant EEPD1 protein and EEPD1 expression levels. (**A-D**) Recombinant EEPD1 wild-type protein was affinity purified using a FLAG-tag and protein preparations were characterized by Coomassie gel staining and western analysis. EEPD1 was purified to a single band of the correct size on gel staining and on western analysis. FEN1 and APE1 were not detected in any EEPD1 protein preparation upon prolonged exposure in western analysis. (**E**) EEPD1 is expressed at significant levels in most tested cell lines. The plot shows EEPD1 levels normalized to β-actin to compare relative EEPD1 abundance among cell lines. HCT116 and HEK293 cells were chosen for these studies because they have relatively high levels of EEPD1. (**F**) EEPD1 protein expression is increased in a dose-dependent manner with increasing concentrations of H_2_O_2_, but prolonged exposure to H_2_O_2_ decreases EEPD1 expression, perhaps because of cell death and/or excessive damage blocking transcription.


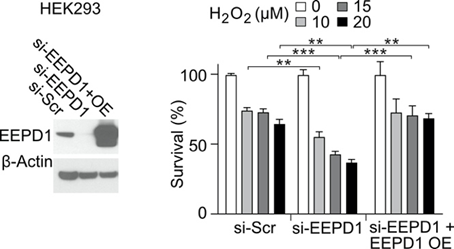


**Figure S3.** Overexpression of WT EEPD1 in si-EEPD1 knockdown restores H_2_O_2_ resistance in HEK293 cells. Western blots and cell survival with si-Scr control, or si-EEPD1 with or without complementation with siRNA-resistant EEPD1 after H_2_O_2_ exposure, as described in Fig. 1. Data plotted as bar graph to show statistical comparisons among different genetic backgrounds.


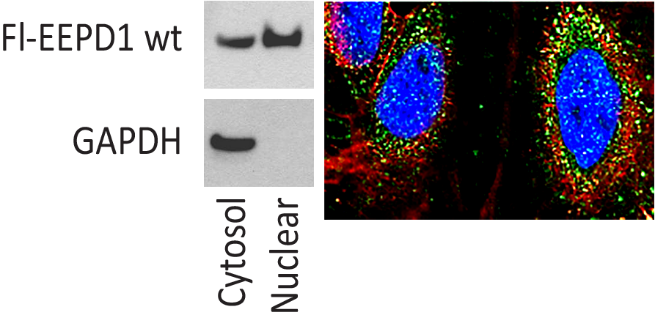


­

**Figure S4. Cellular distribution of EEPD1.** EEPD1 is present in cytosol, nucleus, and membrane. Immunofluorescence image from A549 cells shows EEPD1 (green) localized in the cytosol and nucleus (blue- DAPI stained nucleus) (red- actin). The monoclonal EEPD1 antibody was generated using the epitope described in (26).

**Fig S5. The 5’ endonuclease Metnase is not involved in the repair of oxidatively damaged forks.** (**A**) A strategy used to analyze repair and restart of replication forks after oxidative stress in HEK293 cells with EEPD1 or Metnase siRNA depletion. (**B**) DNA fibers after oxidative stress of Metnase-depleted cells showed that the fraction of stopped forks or restarted replication forks after H_2_O_2_ stress were unaffected by the presence or absence of Metnase. (**C**) Quantitative representation of DNA fiber analysis shown as a percentage of stopped or restarted forks at indicated time points after release from H_2_O_2_. Data are means ± SE from three independent experiments.


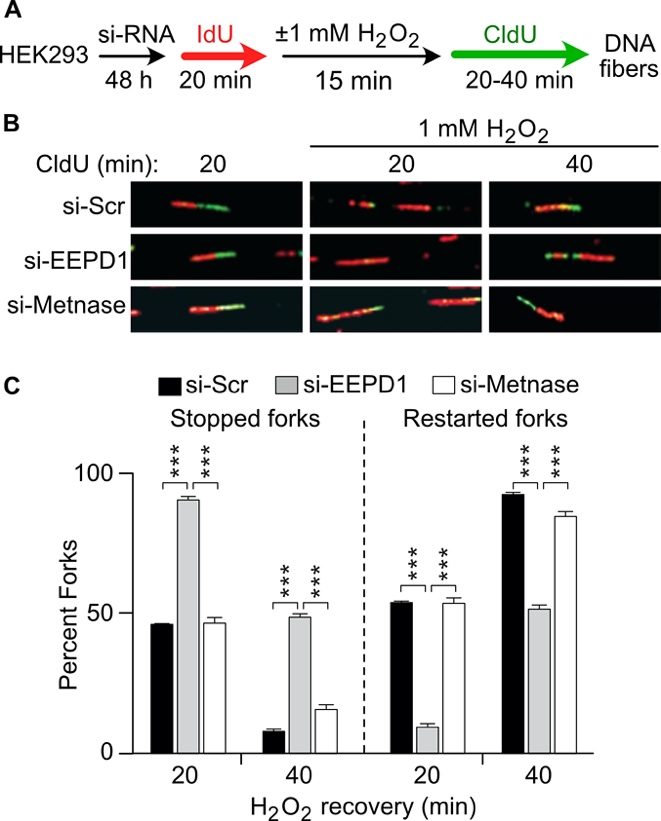


**
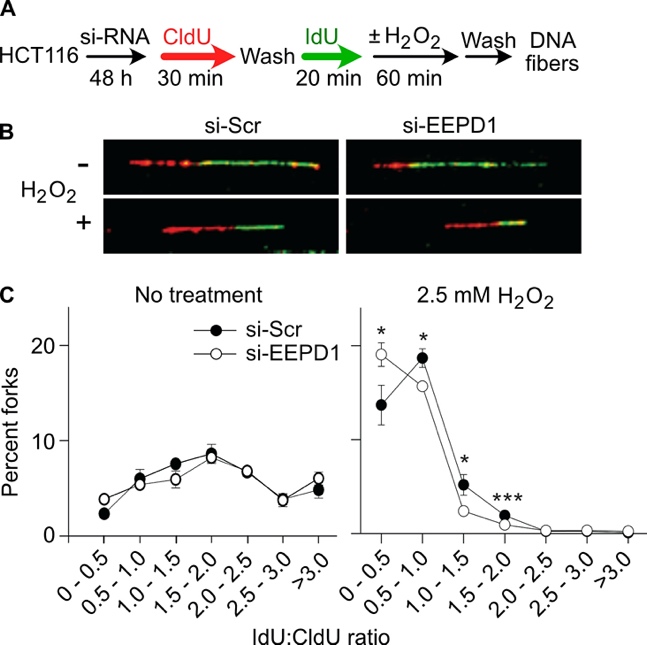
**

**Figure S6.** EEPD1 prevents fork degradation. (**A**) Experimental strategy for the analysis of fork degradation after oxidative stress (1 mM H_2_O_2_) in HCT116 cells depleted of EEPD1. (**B**) Representative images of degrading forks in cells with or without EEPD1 in the presence of continuous H_2_O_2_. (**C**) Quantitation of fork degradation after oxidative stress in cells with or without EEPD1. The decreased ratio of IdU:CIdU (new:old) track lengths on the same fiber in the continued presence of H_2_O_2_ measures fork degradation. Data are from four independent experiments with >200 tracks scored per condition in each experiment. Images of DNA fibers were analyzed by ImageJ software. Data are representative of four independent experiments and plotted as mean ± SE. EEPD1 may prevent fork degradation by promoting HR-mediated fork restart, analogous to the role of BRCA2 in both HR and fork protection.


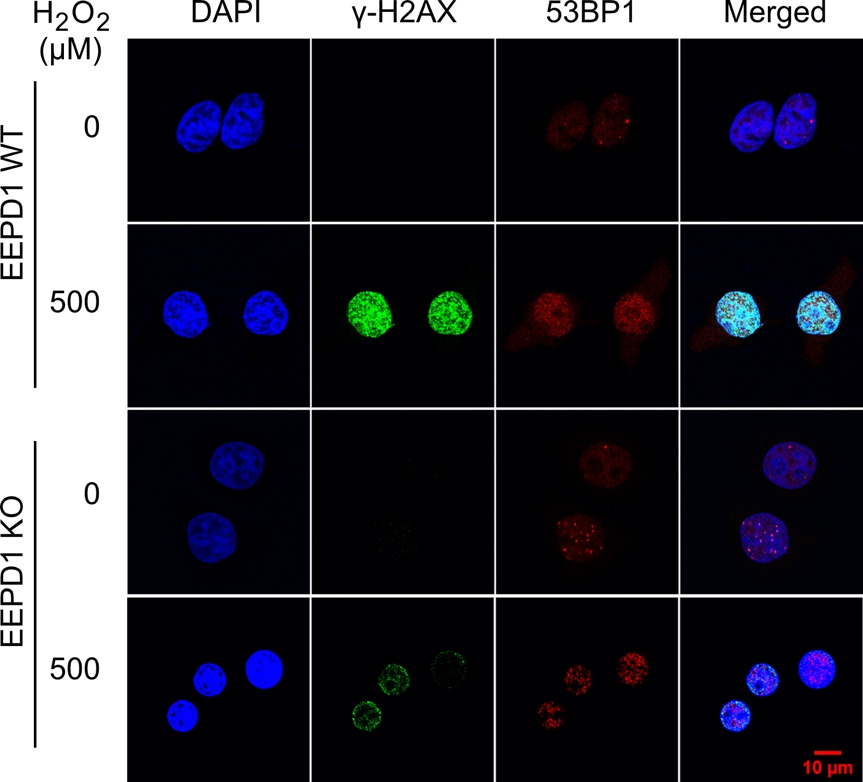


**Figure S7. EEPD1 promotes DNA damage foci formation after oxidative stress.**  γ-H2AX and 53BP1 foci in DAPI-stained nuclei were detected by immunofluorescence microscopy in HeLa control and EEPD1 KO cells during oxidative stress or without such stress, consistent with (26).

**Table S1: Oligonucleotides**

| Oligo A (Sense F DNA) | 5’-FTAGACTAGATGCCTGCAGCTGATG[THF]CGCCGTACGGATCCACGT |
| --- | --- |
| Oligo B (Sense U DNA) | 5’-FTAGACTAGATGCCTGCAGCTGATGUCGCCGTACGGATCCACGT |
| Oligo C (Anti-Sense) | 5’-ACGTGGATCCGTACGGCGGCATCAGCTGCAGGCATCTAGTCTA |
| Oligo D (EMSA_Sense) | 5’- CATGCAGCTGATGCGTACAACACACTGTG |
| Oligo E (EMSA_Anti-Sense) | 5’-CACAGTGTGTTGTACGCATCAGCTGCATG |
| Oligo F (EMSA_fork_Anti-Sense) | 5'-TTTTCCCCCCCCCCCGCATCAGCTGCATG |
| Oligo G (EMSA_fork_Sense Comp) | 5'-CACAGTGTGTTGTA |
| Oligo H (EMSA_fork_Anti-sense Comp) | 5’-GGGGGGGGGGAAAA |
| Oligo I (EMSA Sense F DNA) | 5’- CATGCAGCTGATGC[THF]TACAACACACTGTG |

THF: 3-hydroxy-2-hydroxymethyltetrahydrofuran

F: 6-fluorescein amidite

**Table S2:** **Antibodies and Reagents**

| **Antibody/Protein** | **Supplier** | **Cat No** | **Working dilutions*** |
| --- | --- | --- | --- |
| APE1 | Abcam | AB194 | 1:2000 |
| β-Actin | Sigma | A2228-200 | 1:4000 |
| DNA Polymerase β | Abcam | AB26343 | 1:1000 |
| DNA Ligase 1 | Bethyl | A301-136A | 1:1500 |
| EEPD1 | Produced in lab | -- | 1:1000 to 1500 |
| FEN1 | Bethyl | A300-255A | 1:2000 |
| PARP1 | Cell Signaling | 9532 | 1:2000 |
| PCNA | Abcam | AB29 | 1:1500 |
| Anti-V5 antibody | Life Technology | R960-CUS | 1:2000 |
| Rat-anti-BrdU | Abcam | AB6326 | 2:25 |
| Mouse-anti-BrdU | BD Biosciences | 347583 | 4:25 |
| Anti-Mouse HRP-linked | Cell Signaling | 7076 | 1:3000 |
| Anti-Rabbit HRP-linked | Cell Signaling | 7074 | 1:3000 |
| Goat anti-Rat Alexa fluor594 | Thermo Fisher | A11007 | 1:25 |
| Goat anti-mouse Alexa fluor488 | Thermo Fisher | A21121 | 1:25 |

*Antibody dilutions are approximate and vary from lot to lot of the supplier. Dilutions were linearly optimized for each lot.
